# A kinase-dead *Csf1r* mutation associated with adult-onset leukoencephalopathy has a dominant inhibitory impact on CSF1R signalling

**DOI:** 10.1242/dev.200237

**Published:** 2022-03-25

**Authors:** Jennifer Stables, Emma K. Green, Anuj Sehgal, Omkar L. Patkar, Sahar Keshvari, Isis Taylor, Maisie E. Ashcroft, Kathleen Grabert, Evi Wollscheid-Lengeling, Stefan Szymkowiak, Barry W. McColl, Antony Adamson, Neil E. Humphreys, Werner Mueller, Hana Starobova, Irina Vetter, Sepideh Kiani Shabestari, Matthew M. Blurton-Jones, Kim M. Summers, Katharine M. Irvine, Clare Pridans, David A. Hume

**Affiliations:** 1 Mater Research Institute-University of Queensland, Translational Research Institute, Brisbane, Qld 4102, Australia; 2 Centre for Inflammation Research and Simons Initiative for the Developing Brain, University of Edinburgh, Edinburgh EH16 4TJ, UK; 3 Toxicology Unit, Institute of Environmental Medicine, Karolinska Institutet, Stockholm 171 77, Sweden; 4 Luxembourg Centre for Systems Biomedicine, Université du Luxembourg, Belvaux, L-4401, Luxembourg; 5 UK Dementia Research Institute, Centre for Discovery Brain Sciences, University of Edinburgh, Edinburgh EH8 9XD, UK; 6 Genome Editing Unit, Faculty of Biology, Medicine and Health, University of Manchester, Manchester M13 9PT, UK; 7 Institute for Molecular Biosciences & School of Pharmacy, University of Queensland, Brisbane, Qld 4072, Australia; 8 Department of Neurobiology & Behavior, University of California, Irvine, CA 92697, USA

**Keywords:** CSF1R, Macrophage, Kinase-dead, Leukoencephalopathy

## Abstract

Amino acid substitutions in the kinase domain of the human *CSF1R* gene are associated with autosomal dominant adult-onset leukoencephalopathy with axonal spheroids and pigmented glia (ALSP). To model the human disease, we created a disease-associated mutation (pGlu631Lys; E631K) in the mouse *Csf1r* locus. Homozygous mutation (*Csf1r*^E631K/E631K^) phenocopied the *Csf1r* knockout, with prenatal mortality or severe postnatal growth retardation and hydrocephalus. Heterozygous mutation delayed the postnatal expansion of tissue macrophage populations in most organs. Bone marrow cells from *Csf1r*^E631K/+^mice were resistant to CSF1 stimulation *in vitro*, and *Csf1r*^E631K/+^ mice were unresponsive to administration of a CSF1-Fc fusion protein, which expanded tissue macrophage populations in controls. In the brain, microglial cell numbers and dendritic arborisation were reduced in *Csf1r*^E631K/+^ mice, as in patients with ALSP. The microglial phenotype is the opposite of microgliosis observed in *Csf1r^+/−^* mice. However, we found no evidence of brain pathology or impacts on motor function in aged *Csf1r*^E631K/+^ mice. We conclude that heterozygous disease-associated *CSF1R* mutations compromise CSF1R signalling. We speculate that leukoencephalopathy associated with dominant human *CSF1R* mutations requires an environmental trigger and/or epistatic interaction with common neurodegenerative disease-associated alleles.

## Abstract (precis)

**Summary:** *Csf1r* mutation negatively impacts CSF1R signalling in postnatal development and growth factor responsiveness of macrophage-lineage cells in mice, mirroring the phenotype of adult-onset leukoencephalopathy associated with dominant human *CSF1R* mutations.

## INTRODUCTION

The colony-stimulating factor 1 receptor gene (*Csf1r*) encodes a ligand-dependent tyrosine kinase receptor that controls the survival, proliferation and differentiation of mononuclear phagocyte populations throughout the body, including microglia in the brain (Chitu and Stanley, 2017; Stanley and Chitu, 2014). CSF1R is expressed exclusively in cells of the mononuclear phagocyte lineage, bone marrow (BM) progenitors, monocytes, macrophages and dendritic cells (Grabert et al., 2020) and has two ligands, CSF1 and interleukin 34 (IL-34) (Lelios et al., 2020). Upon ligand binding, CSF1R dimerisation and autophosphorylation generate phosphotyrosine motifs that act as docking sites for multiple downstream effector pathways (Chitu and Stanley, 2017; Stanley and Chitu, 2014). Biallelic recessive loss-of-function mutations in mouse, rat and human *CSF1R* are causally linked to osteopetrosis and postnatal developmental abnormalities (reviewed by Chitu et al., 2021; Hume et al., 2020). In 2011, Rademakers et al. (2011) reported heterozygous amino acid substitutions in the tyrosine kinase domain of CSF1R in patients with autosomal dominant adult-onset leukoencephalopathy with axonal spheroids and pigmented glia (ALSP), now also called CSF1R-related leukoencephalopathy (CRL) (Chitu et al., 2021). Since then, more than 100 different disease-associated *CSF1R* coding mutations have been identified (Chitu et al., 2021; Guo and Ikegawa, 2021; Konno et al., 2018, 2017). Characteristic features of ALSP include enlarged ventricles, cerebral atrophy, periventricular calcifications and thinning of the corpus callosum (Konno et al., 2018, 2017). In ALSP brains, the microglia are reduced in number and altered in their morphology and gene expression (Kempthorne et al., 2020; Tada et al., 2016).

To understand the molecular basis of ALSP, we transfected factor-dependent Ba/F3 cells with expression vectors encoding either wild-type (WT) CSF1R or disease-associated mutant receptors (Pridans et al., 2013). The mutant CSF1R proteins were expressed on the cell membrane at similar levels to WT CSF1R and bound and internalised CSF1 but were unable to support CSF1-dependent cell survival or proliferation. These findings were taken as support for a dominant-negative model for ALSP. In patients, inactive homodimers and heterodimers may also compete with the functional receptor dimers for ligands (Hume et al., 2020). There are obvious parallels with the dominant impact of kinase mutations in the closely related *Kit* in mice and in human piebaldism (Oiso et al., 2013; Reith et al., 1990). Other authors have proposed, based upon analysis of a heterozygous *Csf1r* knockout (*Csf1r*^+/−^) in C57BL/6J mice, that the dominant inheritance in ALSP arises from CSF1R haploinsufficiency (Arreola et al., 2021; Biundo et al., 2020; Chitu et al., 2020, 2015). However, the *Csf1r*^+/−^ model is associated with microgliosis. Neither the microgliosis phenotype nor changes in microglia-specific gene expression was replicated in *Csf1r*^+/−^ rats (Patkar et al., 2021a). Similarly, there is no reported evidence of neuropathology in aged obligate carriers of recessive *CSF1R* loss-of-function alleles in humans (Guo et al., 2019; Guo and Ikegawa, 2021).

Microglia have been ascribed numerous roles in brain development, maturation and synaptic plasticity and in neurodegeneration (Bennett et al., 2018; Favuzzi et al., 2021; Lehrman et al., 2018; Schafer et al., 2012; Wallace et al., 2020) (reviewed by Bohlen et al., 2019; Prinz et al., 2019; Zengeler and Lukens, 2021). Against that background, the generation of a hypomorphic *Csf1r* mutation in mice, which were entirely microglia deficient but developed normally, was surprising (Rojo et al., 2019). The *Csf1r*^ΔFIRE^ mutation removed a conserved intronic enhancer required for expression of the receptor in microglia. *Csf1r*^ΔFIRE/ΔFIRE^ mice also lacked CSF1R expression in BM progenitors and blood monocytes and were deficient in resident macrophage populations in skin, peritoneum, kidney and heart (Rojo et al., 2019). These observations indicate that microglia and certain peripheral macrophage populations are uniquely dependent upon CSF1R and/or have distinct transcriptional regulation.

Konno et al. (2014) reported that transient overexpression of kinase-dead mutant CSF1R proteins in HEK293 cells stably expressing WT CSF1R did not inhibit CSF1-induced autophosphorylation. This finding has been cited as evidence against a dominant-negative model (Chitu et al., 2021), but it is not clear that the mutant and WT proteins were expressed at the same levels in the same cells and at levels comparable to expression in macrophages. Here, we describe the generation and characterisation of mice carrying a germ-line disease-associated *Csf1r*-E631K (pGlu631Lys) mutation, one of the kinase-dead mutations analysed previously in Ba/F3 cells (Pridans et al., 2013). The numbering of amino acids in the mouse and human CSF1R proteins differs; this mutation is orthologous to the human Glu633Lys mutation, originally described in an extended ALSP pedigree (Rademakers et al., 2011). The results confirm the dominant genetic effect of the kinase-dead mutation on CSF1R signalling and provide insights into macrophage homeostasis and the limitations of mice as models of microglial homeostasis.

## RESULTS

### Generation of C57BL/6J.Csf1r^Em1Uman^ (Tg16) mice

To create C57BL/6J.Csf1r^Em1Uman^ (Tg16) mice, guide (g)RNAs and Cas9 were microinjected as described previously (Grabert et al., 2020). Nine founder mice (*Csf1r*^+/E631K^) were crossed with C57BL/6JCrl mice and the offspring interbred. In the initial analysis, undertaken in Edinburgh, the frequencies of *Csf1r*^E631K/E631K^ embryos up to embryonic day (E) 14.5 were not significantly divergent from Mendelian expectation, but no live homozygous pups were obtained. *Csf1r*^E631K/E631K^ embryos were found previously to be almost entirely macrophage and microglia deficient when analysed as controls for the more-selective loss of microglia in *Csf1r*^ΔFIRE/ΔFIRE^ mice (Munro et al., 2020). After transfer of the mutant line to Australia, and cross to the C57BL/6JArc background, the *Csf1r*^E631K^ allele was crossed to the *Csf1r*-EGFP reporter transgenic line (Sasmono et al., 2003) on the same genetic background to enable visualisation of tissue macrophage populations by whole-mount imaging.

**Fig. 1.**
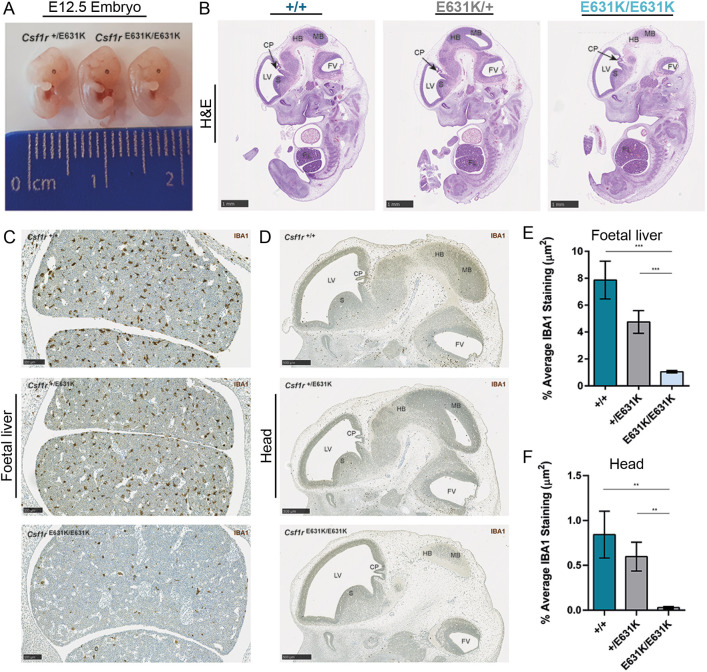
**Effect of *Csf1r*-E631K mutation on the mouse embryo.** (A) Comparison of the gross morphology of a *Csf1r*^E631K/+^ embryo and two *Csf1r*^E631K/E631K^ littermates. (B) Representative midline sections of each genotype stained with H&E. (C,D) Representative IBA1 staining in the foetal liver (C) and E12.5 head (D) of each genotype. (E,F) Morphometric analysis of IBA1 immunolabelling in foetal liver (E) and E12.5 head (F) of each genotype. Graphs shows mean±s.e.m. for *Csf1r*^+/+^ (*n*=3), *Csf1r*^E631K/+^ (*n*=4) and *Csf1r*^E631K/E631K^ (*n*=6). ***P<0.001; **P<0.01 (unpaired Student's *t*-test). CP, choroid plexus; FL, foetal liver; FV, fourth ventricle; HB, hindbrain; LV, left ventricle; MB, midbrain; S, striatum. Scale bars: 1 mm (B); 100 µm (C); 500 µm (D).

*Csf1r* mRNA is expressed in the earliest macrophages produced in the yolk sac (Lichanska et al., 1999) and subsequent development of macrophages in the mouse embryo depends upon CSF1R signalling (Hoeffel et al., 2015; Munro et al., 2020). Fig. 1 shows representative histology and the localisation of embryonic macrophages expressing IBA1 in *Csf1r^+/+^*, *Csf1r*^E631K/+^ and *Csf1r*^E631K/E631K^ embryos at E12.5. The three genotypes were morphologically indistinguishable at this age (Fig. 1A). Histological sections indicated minor developmental delay in the homozygotes, notably in the mid and hind brains (Fig. 1B). Consistent with a complete loss of signalling activity (Pridans et al., 2013) and as reported previously in the context of analysis of a *Csf1r* hypomorphic mutation (Munro et al., 2020), the *Csf1r*^E631K/E631K^ embryos lacked detectable IBA1^+^ macrophages throughout the embryo apart from a small number of IBA1^+^ cells in the liver (Fig. 1C-F). Compared with WT, there was an apparent 30-40% reduction (*P*<0.05) in IBA1^+^ cell populations in the liver and throughout the *Csf1r*^E631K/+^ embryo. CSF1R^+^ macrophages are engaged in active clearance of apoptotic cells during embryonic development, notably between the digits and in the pharyngeal arches, and in removal of expelled erythrocyte nuclei in the liver (Lichanska et al., 1999). However, despite the severe depletion of macrophages in *Csf1r*^E631K/E631K^ mice, we saw no evidence of pyknotic nuclei accumulation.

### The impact of heterozygous *Csf1r*^E631K^ mutation on postnatal development of tissue macrophage populations

**Fig. 2.**
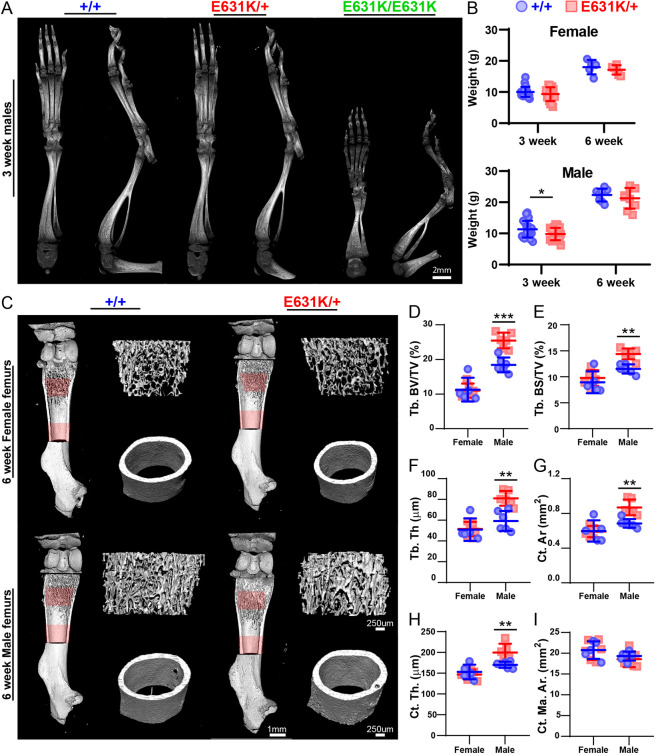
**Effect of heterozygous *Csf1r*-E631K mutation on murine bone development and postnatal growth.** (A) Representative 3D reconstruction of the left hindlimb from micro-CT images of 3-week-old male *Csf1r^+/+^*, *Csf1r*^E631K/+^ and *Csf1r*^E631K/E631K^ mice. (B) Weights of female and male mice at 3 and 6 weeks of age. (C) Representative 3D reconstruction of femurs from micro-CT images of 6-week-old female and male *Csf1r^+/+^* and *Csf1r*^E631K/+^ mice. Scans were performed in 10 μm slices, and a depth of 1500 μm (or 150 slices) was analysed for both the trabecular (Tb) region (start identified as point of fusing of growth plates) and cortical region (start identified as last point of Tb bone). Regions analysed are highlighted in red. (D-I) Micro-CT analysis of (D) percentage Tb bone volume over tissue volume (BV/TV); (E) Tb bone surface-to-volume ratio (BS/TV); (F) Tb thickness (Tb. Th); (G) cortical (Ct.) bone area (Ar); (H) Ct thickness (Ct. Th.); and (I) marrow area in the cortical region analysed (Ct. Ma. Ar.). Data derived from four to six mice of each sex for each genotype at 6 weeks of age. Data are mean±s.d.; *P<0.05, **P<0.001, ***P<0.0001 (unpaired Student's *t*-tests).

On the C57BL/6JCrl background in Edinburgh, no live *Csf1r*^E631K/E631K^ pups resulted from heterozygous mating. The number of *Csf1r*^E631K/+^ pups at weaning was not significantly different from the expected 2:1 ratio of heterozygous:WT. On the C57BL/6JArc background in Australia, occasional *Csf1r*^E631K/E631K^ pups were born. The few live *Csf1r*^E631K/E631K^ mice weighed less than half their littermates by 3 weeks and had severely impaired skeletal development (Fig. 2A). In the *Csf1r*^E631K/+^×*Csf1r*^+/+^ matings, there was no significant loss of *Csf1r*^E631K/+^ pups prior to weaning. Similar to *Csf1r* knockout on the C57BL/6J genetic background (Erblich et al., 2011), *Csf1r*^E631K/E631K^ pups lacked microglia and had severe ventricular enlargement and an almost-undetectable corpus callosum. By contrast, heterozygous mutant (*Csf1r*^E631K/+^) male mice had a small transient lag in postnatal growth (Fig. 2B), but otherwise developed normally, were healthy and fertile and showed no evident behavioural phenotype.

The definitive phenotype of *Csf1* and *Csf1r* mutations in mice and rats is osteoclast deficiency and osteopetrosis (Chitu and Stanley, 2017; Hume et al., 2020). Micro-computed tomography (micro-CT) analysis of adult male and female mice confirmed the substantially lower trabecular bone density and trabecular volume (Fig. 2C,D) that were described previously in female C57BL/6J mice (Sauter et al., 2014). *Csf1r*^E631K/+^ male mice showed a significant increase in trabecular bone density and cortical bone thickness compared with controls, whereas the mutation did not overcome the osteoporosis seen in females (Fig. 2C-I).

**Fig. 3.**
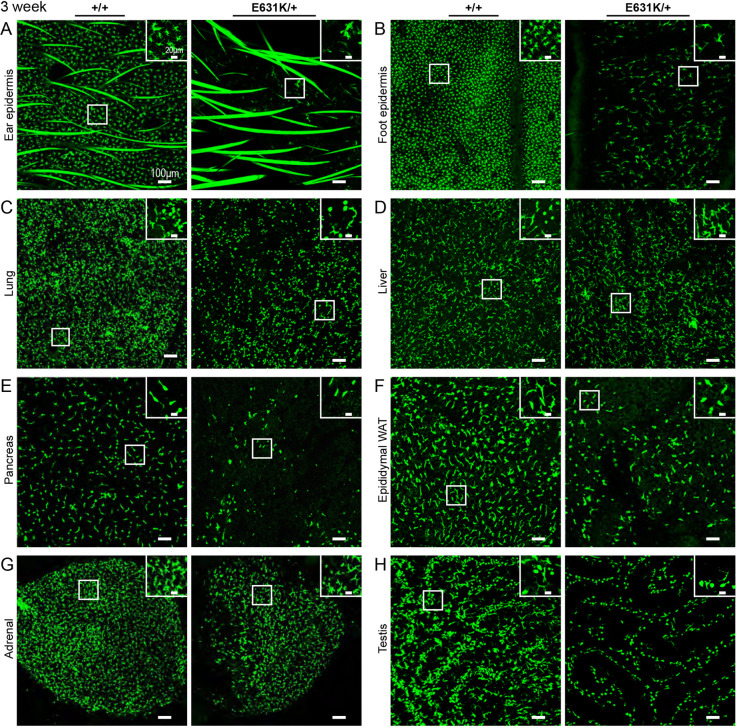
**Effect of heterozygous *Csf1r*-E631K mutation on murine resident tissue macrophage populations at 3 weeks of age.** (A-H) Images of tissues harvested from 3-week-old male *Csf1r-*EGFP transgenic *Csf1r*^+*/+*^ and *Csf1r*^E631K/+^ littermates. Images show the same depth of MIPs for each tissue. The lung (C) and liver (D) projections include stellate subcapsular populations (see insets). Comparable images from 7-week-old males of both genotypes are shown in Fig. S2. Images are representative of at least three mice of each genotype. WAT, white adipose tissue.

**Fig. 4.**
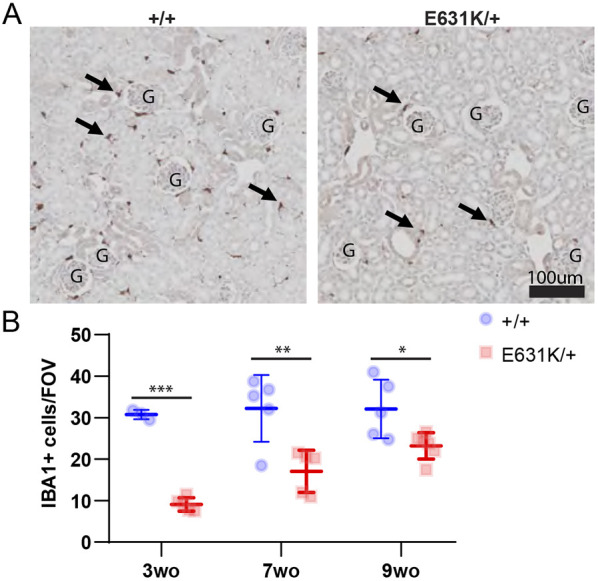
**Age-dependent effect of heterozygous *Csf1r*-E631K mutation on murine resident renal macrophage populations.** (A) Representative IBA1 staining from *Csf1r*^+*/+*^ and *Csf1r*^E631K/+^ kidneys at 3 weeks of age. Arrows indicate IBA1^+^ cells. G, glomeruli. (B) Number of IBA1^+^ cells in the cortex, based on the average number of cells from four field of views (area=0.25 μm^2^) per animal. Data derived from four to six mice of each genotype at 3, 7 and 9 weeks of age. Data are mean±s.d.; *P<0.05, **P<0.001, ***P<0.0001 (unpaired Student's *t*-tests).

Macrophage populations of the mouse and rat expand substantially during the postnatal period. Organs grow rapidly and macrophage density in each organ also increases, as evident from the increase in relative expression of macrophage-expressed transcripts, including *Csf1r* (Summers and Hume, 2017). The relative contribution of the local proliferation of macrophages seeded during embryonic development and postnatal monocyte infiltration varies among individual organs (Ginhoux and Guilliams, 2016; Guilliams et al., 2020; Hume et al., 2019; Yona et al., 2013). The postnatal expansion of the resident mononuclear phagocyte populations is associated with a postnatal increase in *Csf1* mRNA in most organs and is CSF1R dependent, as shown by the impacts of both the *Csf1*^op/op^ and *Csf1r*^–/–^ mutations and the effect of postnatal treatment with anti-CSF1 antibodies (Cecchini et al., 1994; Keshvari et al., 2021a; Rojo et al., 2019; Summers and Hume, 2017; Wei et al., 2005). We predicted that a dominant effect of the *Csf1r*^E631K^ allele on CSF1 responsiveness would compromise and delay this postnatal resident tissue macrophage expansion. As an initial screen for the impact of the mutation on CSF1R-dependent macrophage proliferation, we examined whole mounts using the C*sf1r-*EGFP reporter transgene at 3 weeks and 7 weeks of age. At 3 weeks in *Csf1r*^E631K/+^ mice, *Csf1r-*EGFP-positive macrophages were either absent or greatly reduced in every organ examined. Fig. 3 shows diverse examples: the skin (both ear and foot), adrenal gland, lung, liver, adipose, pancreas and testis. The differences in macrophage density are quantified in Fig. S2. By 7 weeks of age (Fig. S3), most of these tissue macrophage populations appeared indistinguishable between *Csf1r*^E631K/+^ and *Csf1r*^+/+^ littermates. Despite the clear reduction in macrophage density at 3 weeks, the regular spacing that is evident in all tissue-resident populations (Hume et al., 2019) was established. One surprising feature was an apparent difference between Langerhans cell (LC) populations in the ear and footpad. The LC population of the footpad remained absent at 7 weeks, whereas a normal LC density was evident in the ear. To corroborate and quantify the loss of macrophages evident in whole-mount imaging, we located IBA1^+^ macrophages in the cortex of the kidney, which are almost completely macrophage deficient in *Csf1*^op/op^, *Csf1r^−/−^* (Dai et al., 2002) and *Csf1r*^ΔFIRE/ΔFIRE^ mice (Rojo et al., 2019) (Fig. 4A). At 3 weeks, *Csf1r*^E631K/+^ mice had only 30% of the number of renal cortical interstitial IBA1^+^ cells detected in their WT littermates, reaching 53% by 7 weeks and 72% by 9 weeks (Fig. 4B). Despite the delay in macrophage population growth, there was no apparent impact on glomerular or tubular development (Fig. 4A).

**Fig. 5.**
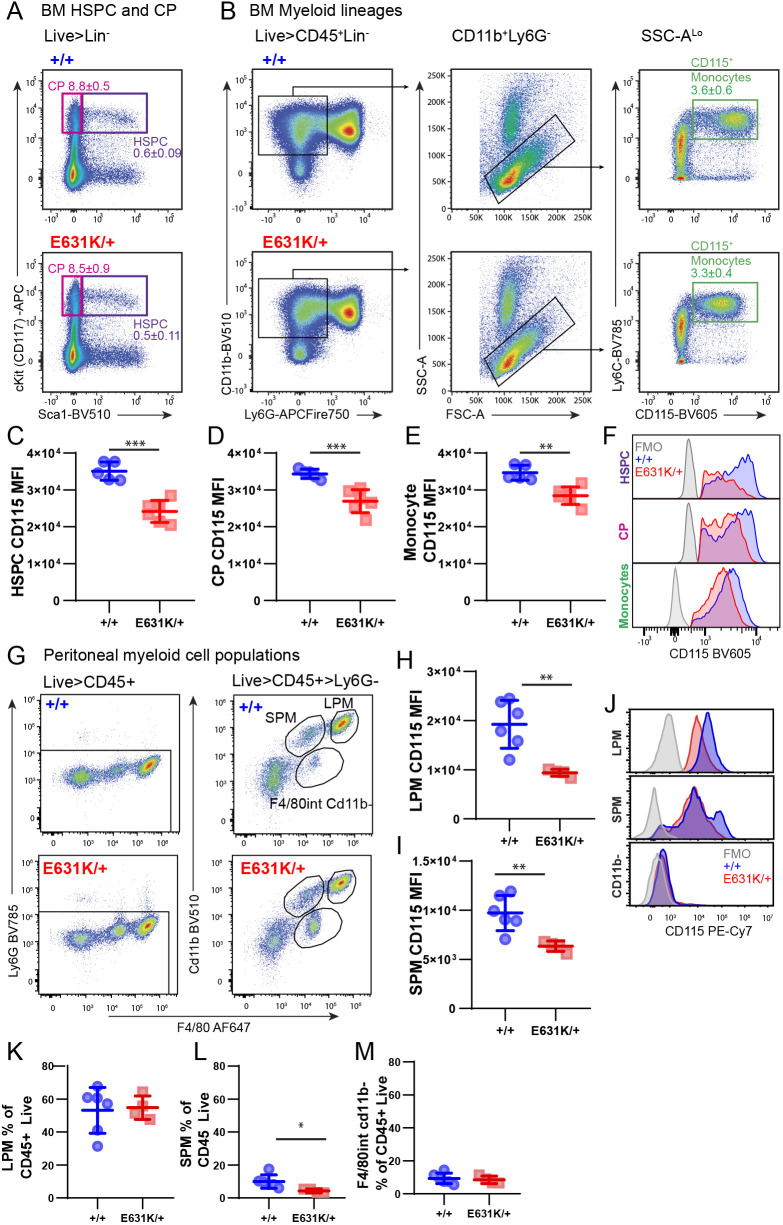
**Impact of heterozygous *Csf1r*-E631K mutation on murine BM and peritoneal macrophage populations and CSF1R expression.** (A-F) Representative flow cytometry plots of (A) HSPCs (purple gate) and CPs (pink gate) and (B) mature myeloid cells of WT (+/+) and E631K heterozygous (E631K/+) mice. Note the left shift of anti-CD115 staining in monocytes in *Csf1r*^E631K/+^ mice (top right panel in B). Inset values are the percentages of these cell populations in proportion to total live cells as in the flow cytometry panel in Table S1. (C-E) CD115 MFIs for the CD115^+^ subset of (C) HSPCs, (D) CPs and (E) Ly6C^+^CD115^+^ monocytes. (F) Overlaid representative histograms (normalised to mode) for each genotype and the fluorescence −1 (FMO) control. (G-J) Flow cytometry analysis of CD45^+^ peritoneal (PT) cells from each genotype. (G) Representative flow cytometry gating strategy of PT cells stained for F4/80 and CD11b. CD115 MFI in (H) LPMs and (I) SPMs. (J) Overlaid representative histograms (normalised to mode) for CD115 staining for each F4/80^+^ population and each genotype and FMO control. (K-M) Proportional quantification of (K) LPMs, (L) SPMs and (M) F4/80^int^CD11b^−^ cells as a percentage of total CD45^+^ cells. Data derived from four to six mice for each genotype at 3 weeks of age. Data are mean±s.d.; *P<0.05, **P<0.001, ***P<0.0001 (unpaired Student's *t*-tests).

Fig. 5A shows a flow cytometry analysis of BM progenitor and monocyte populations in 3-week-old animals. In *Csf1r*^ΔFIRE/ΔFIRE^ mice, CSF1R was undetectable in BM or blood based on binding of anti-CD115 or labelled CSF1-Fc, yet there was no detectable change in BM progenitor populations or blood monocyte profiles (Rojo et al., 2019). Monocyte numbers are also unaffected by anti-CSF1R treatment in adults (MacDonald et al., 2010). Hence, although CSF1 treatment can drive monocyte proliferation *in vitro* and *in vivo*, and local CSF1 is proposed to be a key component of the myelopoietic niche in BM (Zhang et al., 2021), CSF1R signalling is not absolutely required for monocyte production. We analysed hematopoietic stem and progenitor (HSPC), committed progenitor (CP) and monocyte populations using surface markers as previously described (Grabert et al., 2020). Fig. 5A,B shows representative fluorescence-activated cell sorting (FACS) profiles and gating. There was no significant effect of *Csf1r*^E631K/+^ mutation on the abundance of stem and progenitor cells, committed precursors or mature myeloid cell populations compared with controls. In those cells that expressed detectable CD115, the distribution of staining was shifted and the level of expression (mean fluorescence intensity; MFI) on individual cells was reduced in *Csf1r*^E631K/+^ BM populations, with the greatest impact on progenitors (Fig. 5C-F). Fig. 5G-I shows the analysis of peritoneal populations. Whereas the relative abundance of F4/80^hi^ large peritoneal macrophages (LPMs) was unchanged in *Csf1r*^E631K/+^ mice, there was selective reduction of the small F4/80^lo^ peritoneal macrophage subset (SPMs) (Fig. 5L). SPMs are monocyte derived and may serve as precursors for the slow replacement of embryo-derived macrophages (Bain et al., 2016). Peritoneal macrophage populations expressed abundant surface CD115, whereas CD11b^−^ peritoneal cell populations were negative for CD115. The MFI in both LPMs and SPMs was reduced significantly in *Csf1r*^E631K/+^ mice (Fig. 5J).

### The impact of heterozygous *Csf1r*^E631K^ mutation on brain microglial populations, motor functions and pathology

**Fig. 6.**
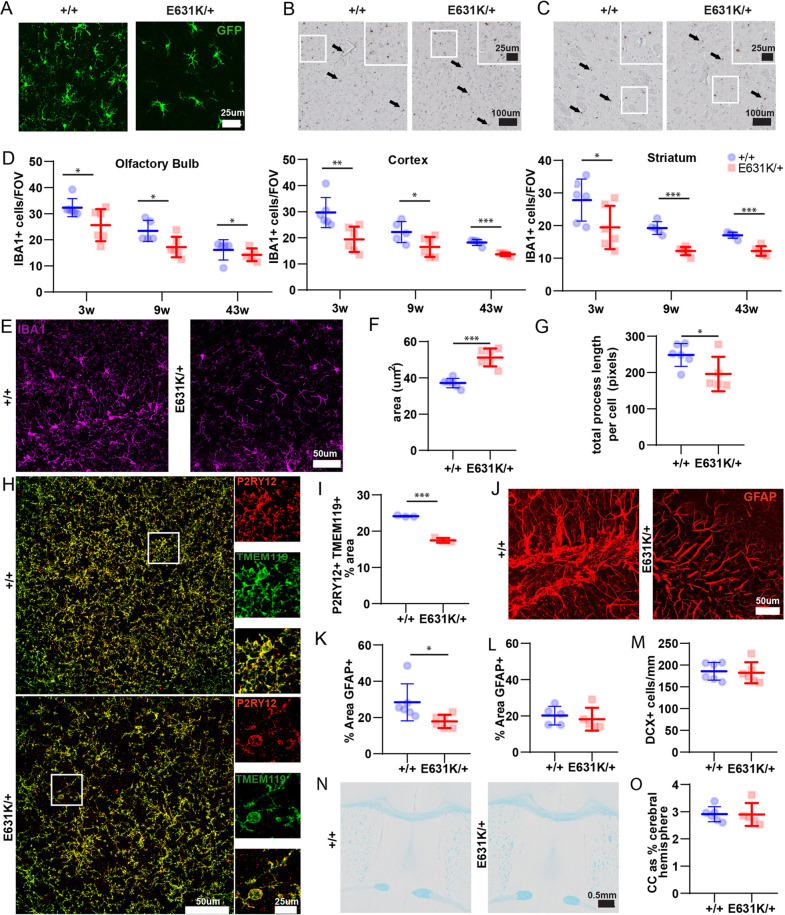
**Effect of heterozygous *Csf1r*-E631K mutation on microglia and other cell populations in the murine brain.** (A) Representative MIPs of confocal *z*-stack series of whole-mount cortex from 7-week-old mice transgenic for *Csf1r*-EGFP. Images show the same depth of MIP. (B,C) Representative IBA1 staining in the cortex (B) and striatum (C) of 9-week-old mice. Individual and merged staining for the insets is shown on the right. (D) Quantitative analysis of IBA1^+^ cells in the olfactory bulb, cortex and striatum of 3-, 9- and 43-week-old mice. (E-G) Analysis of microglia density and morphology. (E) MIP of confocal *z*-stack series showing IF localisation of IBA1^+^ cells in dentate gyrus of 7-week-old mice. (F,G) Quantitative analysis of IBA1^+^ cells in the dentate gyrus performed on microglial cell body area (average of 20 cells/animal) (F) and average total process length per cell (average of three fields of view per animal) (G). (H) Representative MIP of immunofluorescent localisation of P2RY12, TMEM119 and merged staining in the cortex of 7-week-old mice showing complete overlap. (I) Quantitative analysis of the percentage area of colocalised P2RY12 and TMEM119 staining in the cortex of 7-week-old mice. (J) Representative MIP of confocal *z*-stack series showing GFAP staining in the dentate gyrus (DG) of 7-week-old mice. (K,L) Quantitative analysis of the percentage area of GFAP^+^ staining (average of three areas) of 7-week-old brains using immunofluorescence histochemistry (K) and 43-week-old brains using IHC (L). (M) Quantitative analysis of the number of DCX^+^ cells/mm of DG (average of three DG/animal). (N) Representative Luxol Fast Blue with Cresyl Fast Violet counter stain in 9-week-old brains. (O) Quantitative analysis of the area of half of the corpus callosum (CC) as a percentage area of the cerebral hemisphere at 9 weeks of age. *n*=5 or 6/group. Data are mean±s.d.; *P<0.05, **P<0.01, ***P<0.001, (unpaired Student's *t*-tests).

Similar to macrophage populations in the periphery, the microglial population of the mouse brain expands during the postnatal period (Nikodemova et al., 2015; Perry et al., 1985). Imaging using the *Csf1r*-EGFP reporter indicated that, unlike *Csf1r*^ΔFIRE/ΔFIRE^ mice, *Csf1r*^E631K/+^ mice were not globally deficient in microglia, but there was a clear phenotype. A comparison of *Csf1r*-EGFP in cortical microglia in WT and *Csf1r*^E631K/+^ littermates at 7 weeks of age showed that, aside from the decrease in the number of microglial cell bodies in any field, there was an obvious reduction in dendritic arborisation of the membrane processes in the *Csf1r*^E631K/+^ mice (Fig. 6A). To document these changes more thoroughly, we performed IBA1 staining on multiple brain regions at different ages (Fig. 6B-G). These analyses were carried out independently in both Brisbane and Edinburgh, where the mutations were on subtly different C57BL/6J backgrounds. IBA1^+^ microglial numbers in all brain regions in juvenile (3 weeks), young adult (7-9 weeks) and aged (43 weeks) *Csf1r*^E631K/+^ mice were reduced by 20-40% (Fig. 6D), with a downward trend in microglial number with age independent of *Csf1r* genotype, as reported by others (Nikodemova et al., 2015). The data generated independently in Edinburgh from mice at 9 weeks of age for cerebellum and forebrain are shown in Figs S4 and S5. CSF1-dependent microglia have been attributed functions in the development and turnover of Purkinje cells (Kana et al., 2019; Marin-Teva et al., 2004), but there was no evidence of any phenotype in the cerebellum, despite reduced numbers and apparent ramification of microglia in both white and grey matter.

To quantify the effect on microglial arborisation, we analysed images of individual microglia from 7-week-old brains using ImageJ (https://imagej.net/). Microglia in *Csf1r*^E631K/+^ mice had an increased cell body area and greatly reduced ramification of membrane processes (Fig. 6F,G). Such altered morphology is considered an indication of reactive microglia and is a feature of human ALSP (Kempthorne et al., 2020; Tada et al., 2016). In a model of Cre-induced heterozygous mutation of *Csf1r* (Arreola et al., 2021), microglial dyshomeostasis was evident from reduced expression of microglia-enriched markers, such as P2RY12, but this model was also heterozygous for a *Cx3cr1* mutation. Quantification of the overlapping distribution of the homeostatic markers P2RY12 and TMEM119 in *Csf1r*^+/+^ and *Csf1r*^E631K/+^ cortex (Fig. 6H) confirmed the reduced density (Fig. 6I) of microglia seen with IBA1, although the staining intensity and punctate distribution of these markers on individual cells were unaffected by the *Csf1r* mutation.

Microglia have been attributed many functions in postnatal brain development through interactions with neurogenic progenitors, neurons, astrocytes and oligodendrocytes (Han et al., 2021; Matejuk and Ransohoff, 2020; Prinz et al., 2019). Microglia interact with, and regulate the function of, astrocytes, and astrogliosis is a common feature of neuroinflammatory diseases, including ALSP (Han et al., 2021; Matejuk and Ransohoff, 2020). We analysed the distribution of astrocytes and doublecortin (DCX)-positive neurogenic progenitors in the hippocampus of 7-week-old *Csf1r*^E631K/+^ and control mice following GFAP staining (Fig. 6J,K). Within the dentate gyrus, the apparent density of GFAP staining was reduced by almost 40% in 7-week-old *Csf1r*^E631K/+^ mice. In 43-week-old *Csf1r*^E631K/+^ mice, there was no longer any difference in GFAP density compared with controls because of the relative decrease in density in the latter (Fig. 6L). We reported elsewhere that, in *Csf1rko* rats (Patkar et al., 2021a), there was a dysregulation of the differentiation and arborisation of DCX^+^ neurogenic progenitors in the dentate gyrus. However, there was no discernible difference in the number of DCX^+^ neurons in 7-week-old *Csf1r*^E631K/+^ mice (Fig. 6M). Finally, Chitu et al. reported thinning of the corpus callosum and lateral ventricle enlargement in affected mice in the *Csf1r^+/−^* model (Chitu et al., 2015). Although we did not undertake detailed ultrastructural analysis, we saw no evidence of the ventricular enlargement seen in homozygous mutants or any altered development of the corpus callosum in *Csf1r*^E631K/+^ mice (Fig. 6N,O)

The reduced microglial density and altered morphology observed in *Csf1r*^E631K/+^ mice are consistent with human ALSP (Kempthorne et al., 2020; Tada et al., 2016), but not with the increased microglial density reported in *Csf1r*^+/−^ mice on the C57BL/6J background (Arreola et al., 2021; Chitu et al., 2020, 2015). *Csf1r*^ΔFIRE/+^ mice also have a 50% reduction in *Csf1r* mRNA in the juvenile brain, but comparable microglial density as indicated by expression of a suite of microglia-associated transcripts (Rojo et al., 2019). On the original mixed genetic background, we found no impact of the *Csf1r*^ΔFIRE/+^ genotype on the yield or relative abundance of CD45^low^CD11b^+^ microglia up to 9 months of age (Rojo et al., 2019). However, the effects of *Csf1r* mutation in mice are strongly influenced by genetic background (Chitu and Stanley, 2017) and the haploinsufficiency model is specific to C57BL/6J mice. We confirmed that the 50% loss of microglial CSF1R was associated with a 2-fold increase in microglial density in hippocampus, motor cortex and corpus callosum in 6-month-old C57BL/6J *Csf1r*^ΔFIRE/+^ mice compared with WT (Fig. S6). There was also a marginal increase in GFAP^+^ astrocytes in the hippocampus.

**Fig. 7.**
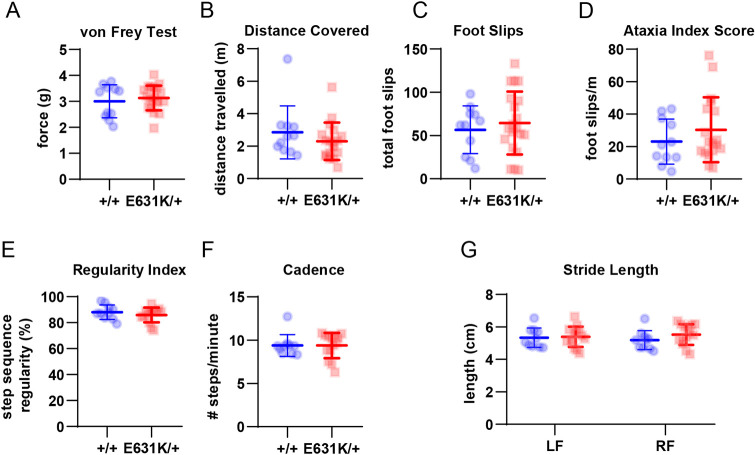
**Effect of *Csf1r* genotype on sensorimotor parameters in aged mice.** (A) Quantification of the Von Frey test as a measurement of mechanical allodynia in rodents. The vertical axis shows the threshold force at which the mouse withdrew its paw, a measure of pain sensitivity. (B-D) Quantification of the parallel rod floor test as a measure of balance and coordination, quantified as distance covered (B), number of foot slips (C) and ataxia index score (D). (E-G) Quantification of the CatWalk XT gait analysis showing results for step regularity index (E), cadence (F) or stride length of either the left front (LF) or right front (RF) paw (G). Data derived from 10-14 mice for each genotype at 43 weeks of age. Data are mean±s.d. No significant differences were detected between genotypes (unpaired Student's *t*-tests).

Many patients with ALSP present initially with sensorimotor deficiencies (Chitu et al., 2021; Konno et al., 2018, 2017) and CSF1R signalling has been directly implicated in neuropathic pain (Saleh et al., 2018). To seek evidence of these symptoms in *Csf1r*^E631K/+^ mice, we performed a range of sensorimotor tests, but were unable to demonstrate any significant impacts of the *Csf1r*^E631K/+^ genotype on gross motor function or mechanical paw withdrawal thresholds measured at 10 months of age (Fig. 7).

To further test for age-dependent effects of the *Csf1r*^E631K/+^ genotype, we also examined an even older cohort (15 months). No periventricular calcification or evidence of pathology were detected by magnetic resonance imaging (Fig. S7A) or upon histological examination of multiple brain regions. Chitu et al. (2020, 2015) emphasised the development of microgliosis in the white matter of *Csf1r*^+/−^ mice as a model of ALSP. IBA1 staining of these aged brains revealed occasional clusters of reactive microglia and apparent microglial heterogeneity in striatum and corpus callosum but with equal prevalence in *Csf1r*^+/+^ and *Csf1r*^E631K/+^ mice. The absolute differences in overall IBA1^+^ cell density seen in younger animals were no longer evident (Fig. S7B). Confirming this conclusion, disaggregation of the aged brains yielded similar numbers of microglia with unchanged profiles of fluorescence intensity of the microglial markers CD11b, CD45 and P2RY12 (Fig. S8).

In summary, the data indicate that the heterozygous *Csf1r* kinase-dead mutation in mice has a dominant inhibitory effect on the development of CSF1R-dependent microglia and selected tissue macrophage populations.

### Dominant repression of CSF1 responsiveness in the periphery in *Csf1r*^E631K/+^ mice

**Fig. 8.**
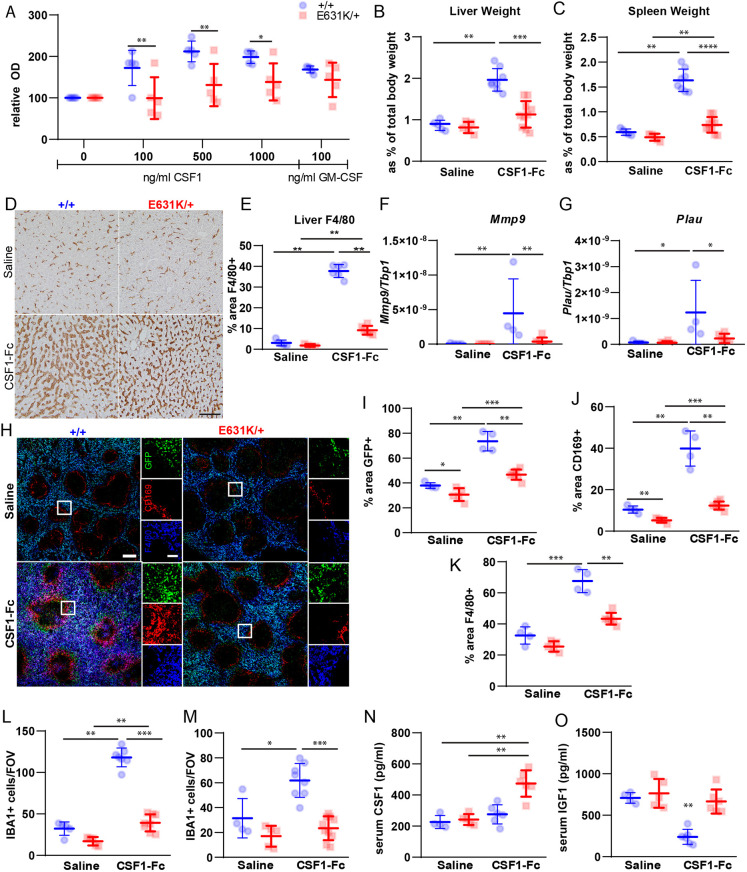
**Effect of heterozygous *Csf1r*-E631K mutation on responses to CSF1 *in vitro* and *in vivo*.** (A) *In vitro* response of BM cells from *Csf1r^+/+^* and *Csf1r^E631K/+^* mice to recombinant human CSF1 or mouse GM-CSF (CSF2), expressed as optical density (OD) relative to the unstimulated cultures in each case. *n*=5/group; four technical replicates per animal. (B-O) *In vivo* response. Weight of the liver (B) and spleen (C) of all animals following acute CSF1-Fc treatment. (D) Representative images of F4/80 IHC staining of liver sections from all treatment groups. (E) Percentage area stained for F4/80 in liver sections (average of four areas/animal). (F,G) qPCR analysis showed upregulation of CSF1R target genes, including *Mmp9* (F) and *Plau* (G). (H) Representative images of GFP, CD169 and F4/80 staining of spleen sections from all treatment groups. (I-K) Quantification of the percentage area stained for GFP (I), CD169 (J) and F4/80 (K) in spleen sections (average of two depths/animal, with least 50% of each spleen on the sagittal sections analysed). (L,M) Quantitative analysis of IBA1^+^ cells in the (L) kidney and (M) heart. (N,O) Serum CSF1 (N) and IGF1 (O) in control and treated mice as indicated; *n*=4-9 per group. *P<0.05, **P<0.01, ***P<0.001, ****P<0.0001 [two-way ANOVA with multiple comparisons (A) or Mann–Whitney U test (B,C,E-G,I-O)]. Scale bars: 200 μm (H); 50 μm (D, inset H).

Whereas there was a relatively mild phenotype in the brain, the heterozygous kinase-dead mutation clearly impacted development of CSF1R-dependent resident macrophage populations in the periphery (Fig. 3). Therefore, we examined whether these impacts could be linked directly to a loss of CSF1 responsiveness. BM cells respond to the addition of CSF1 in liquid culture to generate confluent cultures of adherent macrophages within 5-7 days. In *Csf1r*^ΔFIRE/ΔFIRE^ mice, which lack CSF1R expression in BM progenitors, this *in vitro* proliferative response was greatly reduced, whereas the *Csf1r*^ΔFIRE/+^ marrow response to CSF1 was not significantly different from that of *Csf1r*^+/+^ mice (Rojo et al., 2019). Consistent with the loss of CSF1R in progenitors (Fig. 5), BM cells isolated from *Csf1r*^E631K/+^ mice were almost completely deficient in the proliferation and differentiation response to CSF1 *in vitro*, whereas the response to CSF2 (GM-CSF) was unaffected (Fig. 8A).

To determine whether the CSF1 resistance *in vitro* also occurred *in vivo*, we examined the response to administration of exogenous CSF1. Treatment of mice with a pig CSF1-Fc fusion protein caused monocytosis, expansion of tissue macrophage populations through monocyte recruitment and local proliferation and consequent hepatosplenomegaly (Gow et al., 2014). Here, we used a human CSF1/mouse Fc protein that has similar biological activity to the pig CSF1-Fc fusion protein and that was generated for preclinical evaluation. Fig. 8B-O compares the responses of *Csf1r*^E631K/+^ mice and *Csf1r*^+/+^ controls to acute administration of a maximal dose of CSF1-Fc on four successive days with analysis on day 5. The monocytosis, increased size of the liver and spleen and expansion of F4/80^+^ macrophage populations observed in controls in response to CSF1-Fc were almost entirely absent in *Csf1r*^E631K/+^ mice (Fig. 8B-E). Analysis of liver mRNA confirmed the induction of the CSF1R target genes, *Plau* and *Mmp9* (Gow et al., 2014) in controls, which was undetectable in *Csf1r*^E631K/+^ mice (Fig. 8F,G). One previously unknown circulating biomarker of the response to CSF1-Fc is the somatic growth factor, insulin-like growth factor 1 (IGF1), which was reduced to almost undetectable levels in serum from treated *Csf1r*^+/+^ mice (Fig. 8O), likely associated with proliferative expansion of the liver (Gow et al., 2014). IGF1 in serum was unaffected by CSF1-Fc in *Csf1r*^E631K/+^ mice (Fig. 8O). Treatment with human CSF1-Fc effectively competed with the endogenous ligand and caused a transient increase in circulating mouse CSF1 that was resolved by day 5 as a result of expansion of tissue macrophages and consequent CSF1 clearance. Consistent with the lack of efficacy in *Csf1r*^E631K/+^ mice, endogenous CSF1 remained elevated in these mice on day 5 (Fig. 8N).

The CD169^+^ marginal zone macrophage population in spleen is particularly CSF1/CSF1R dependent in both mouse and rat (Pridans et al., 2018; Witmer-Pack et al., 1993). Consistent with the selective loss of CSF1R-dependent macrophages, this population remained greatly reduced in untreated adult *Csf1r*^E631K/+^ spleen, whereas the F4/80^+^/*Csf1r*-EGFP^+^ cells of the red pulp were less affected (Fig. 8H-K). Splenomegaly in CSF1-Fc-treated *Csf1r*^+/+^ mice was associated with both expansion of the marginal zone CD169^+^ populations and extensive expansion of the CD169^+^ populations in the red pulp. Both responses were prevented in *Csf1r*^E631K/+^ mice (Fig. 8J). As noted above, *Csf1r*^E631K/+^ mice had reduced numbers of macrophages in kidney and heart even as adults. CSF1-Fc treatment expanded the IBA1^+^ macrophage populations in both organs in *Csf1r*^+/+^ mice but had no effect in heterozygous *Csf1r*^E631K/+^ mice (Fig. 8L,M).

## DISCUSSION

In this study, we analysed the effect of a germline kinase-dead *Csf1r* mutation (*Csf1r*^E631K^), equivalent to the E633K mutation associated with human ALSP, on the mouse mononuclear phagocyte system. The homozygous E631K mutation phenocopied the impact of a homozygous null mutation (*Csf1r*^−/−^; Chitu and Stanley, 2017; Erblich et al., 2011), confirming that the mutation abolishes signalling activity. Similar to the knockout mutation on the C57BL/6J background, homozygous mutant (*Csf1r*^E631K/E631K^) embryos lacked macrophages (Fig. 1). The few pups that survived embryonic development and were born had severe postnatal growth retardation and hydrocephalus.

The postnatal developmental phenotype in *Csf1r*^E631K/+^ mice is consistent with a partial loss of CSF1R activity. *Csf1r*^E631K/+^ mice had a reduced postnatal growth rate, mild male osteopetrosis and delayed development of tissue macrophage populations (Figs 2 and 3, Fig. S3). The tissues impacted by *Csf1r*^E631K/+^ mutation include the lung, in which CSF2 (GM-CSF) is the major growth factor required for the development of alveolar macrophages and homeostasis (Guilliams et al., 2013), whereas CSF1 is required for postnatal expansion of resident macrophages (Jones et al., 2014 and references therein). The CD169^+^ marginal zone macrophages of spleen, which are entirely depleted in both *Csf1*^op/op^ mice and *Csf1r*^−/−^ rats (Pridans et al., 2018; Witmer-Pack et al., 1993), remained reduced even in adult *Csf1r*^E631K/+^ mice. The regular distribution of *Csf1r-*EGFP^+^ cells in every tissue in juvenile *Csf1r*^E631K/+^ mice despite their reduced density (Fig. 3) supports a model in which macrophages occupy territories that are established by mutual repulsion rather than by precisely defined spatial niches (Hume et al., 2019).

The analysis of the response to CSF1 in BM cells *in vitro* provides unequivocal evidence that the disease-associated mutation has a dominant inhibitory effect on CSF1R signalling when expressed at normal physiological levels in natural target cells. The level of surface CSF1R detected with anti-CD115 antibodies was reduced in BM progenitors in *Csf1r*^E631K/+^ compared with *Csf1r*^+/+^mice. Whereas disease-associated mutations did not prevent expression of CSF1R on the cell surface in transfected factor-dependent BaF3 cells (Pridans et al., 2013), the most common human mutation (I794T) compromised ectodomain shedding of CSF1R overexpressed in HEK293 cells (Wei et al., 2021). A dominant impact of the mutant protein on the stability of WT CSF1R cannot be excluded. However, the selective loss of CSF1R in marrow progenitors compared with monocytes (Fig. 5F) might also be a consequence of a signalling defect, because *Csf1r* mRNA and protein expression is CSF1 inducible in progenitors and increases during differentiation (Grabert et al., 2020; Tagoh et al., 2002).

Regardless of the underlying mechanism, *Csf1r*^E631K/+^ mice are not entirely CSF1R deficient, because all CSF1R-dependent resident macrophages that can be depleted rapidly by anti-CSF1R antibodies (MacDonald et al., 2010) are present in adults. However, adult *Csf1r*^E631K/+^ mice were CSF1 resistant *in vivo*. Consistent with previous studies with pig CSF1-Fc (Gow et al., 2014; Stutchfield et al., 2015), the human–mouse CSF1-Fc used in the current study promoted monocytosis and proliferative expansion of the liver and spleen and increased macrophage populations in multiple organs. We showed that CSF1-Fc treatment is associated with, among other impacts, a rapid fall in circulating IGF-1. We extended earlier data (Gow et al., 2014) to demonstrate the expansion of macrophage populations in the heart and kidney (organs in which tissue macrophages are particularly CSF1R dependent; Rojo et al., 2019) and the observation that CSF1 selectively expands CD169^+^ populations in both the marginal zone and red pulp of spleen. Mechanistic studies of the pleiotropic effects of CSF1-Fc administration are ongoing. The key finding herein is that all these responses to CSF1-Fc administration were reduced or abolished in *Csf1r*^E631K/+^ mice.

The number of microglia in all regions of the brain of embryo, juvenile and adult *Csf1r*^E631K/+^ mice was reduced by 20-40%. A more striking impact was reduced microglial spreading and ramification, which may reflect the well-studied ability of CSF1 to promote membrane ruffling, filipodia formation and spreading on a substratum (Stanley and Chitu, 2014). These mouse microglial phenotypes closely resembled those reported in patients with ALSP (Kempthorne et al., 2020; Tada et al., 2016). We have also generated mice with the equivalent to the most-common human ALSP-associated mutation, *Csf1r*^I794T^, which was also signalling deficient when expressed in Ba/F3 cells (Pridans et al., 2013). Preliminary analysis of these mice supports the partial microglial deficiency described in *Csf1r*^E631K/+^ mice. The microglial difference between *Csf1r*^E631K/+^ and *Csf1r*^+/+^ mice was no longer evident at 15 months. However, the data indicate that this convergence was the result of a progressive reduction in microglial density in the controls rather than of recovery in the mutant (Fig. 6). There was no evidence of functional deficits or overt brain pathology resembling the human disease. The delay in microglial population and phenotypic modulation in younger animals was associated with a delay in astrocyte development, which also recovered with age (Fig. 6). There are numerous trophic interactions between microglia and astrocytes (Matejuk and Ransohoff, 2020) that likely contribute to this link. Importantly, in the older animals, there was no evidence of astrocytosis, which is commonly seen in neuroinflammation.

The phenotype of *Csf1r*^E631K/+^ mice was clearly different from the reported phenotype of *Csf1r*^+/−^ mice (Chitu et al., 2020, 2015, 2021), although the effects on peripheral macrophage populations and CSF1 responsiveness have not been analysed in the haploinsufficiency model. Without a direct comparison on the same genetic background, we cannot conclude that the effect of the heterozygous kinase-dead mutation is greater than the effect of a null mutation. However, the *Csf1r*^ΔFIRE^ mutation, similar to the complete knockout, is not dosage compensated (Rojo et al., 2019). Accordingly, as in the complete knockout in heterozygotes, there was a 50% reduction in CSF1R on individual microglia and on BM progenitors and monocytes, whereas CSF1 responsiveness *in vitro* was not affected (Rojo et al., 2019). We were able to recapitulate the increased microglial density reported in aged C57BL6/J *Csf1r*^+/−^ mice (Chitu et al., 2020, 2015, 2021) in heterozygous *Csf1r*^ΔFIRE^ mice on the same C57Bl/6J background (Fig. S5). We suggest that, at least on this genetic background, the 50% loss of expression has a minimal impact on proliferation but reduces CSF1R-mediated endocytosis, allowing growth factors to increase in the brain with time. Accordingly, with age, a denser population of microglia can be maintained by the available CSF1 and IL34. Interestingly, De et al. (2021, 2014) described a model of microgliosis associated with transgenic expression of CSF1 in astrocytes that did not give rise to overt neuropathology.

Neither microglia-deficient *Csf1r*^ΔFIRE/ΔFIRE^ nor CSF1-resistant *Csf1r*^E631K/+^ mice exhibited the brain pathology associated with the loss of microglia resulting from dominant and homozygous recessive CSF1R mutations in human patients. However, it is not clear whether CSF1R mutations are strictly causal in human ALSP. There are multiple reports of asymptomatic aged individuals who carry kinase-dead mutations that are disease associated in their progeny, siblings or other relatives (reviewed by Chitu et al., 2021). CSF1R mutations might interact genetically with other common allelic variants associated with susceptibility to neurodegeneration. There is certainly evidence of epistatic interactions between *Csf1r* mutations and genetic background in mouse strains, with C57BL/6J mice being uniquely sensitive (Chitu et al., 2016). There are obvious parallels with another adult-onset microgliopathy, Nasu–Hakola disease, which is associated with mutations in *TREM2* or the adaptor, *TYROBP*, which lie downstream of CSF1R in regulation of microglial survival (Otero et al., 2009). *Trem2*^−/−^ mice do not exhibit neurodegenerative pathology but TREM2 loss-of-function sensitises the carrier to the development of disease in dementia models (Filipello et al., 2018; Ulland et al., 2017).

No mouse model can recapitulate the gene-by-environment interactions that are well documented in more-common forms of neurodegenerative disease (Dunn et al., 2019) and likely also influence disease progression in ALSP. There are numerous reports of the roles of microglia in neuroprotection (reviewed by Bohlen et al., 2019; Prinz et al., 2019; Zengeler and Lukens, 2021). An inability to respond to CSF1/IL34 in the brain and/or in the periphery could predispose to brain pathology. For example, the early CSF1-dependent microglial response is essential for efficient control of Herpes simplex virus encephalitis challenge in mice (Uyar et al., 2020) and microglia are implicated in many aspects of physiological myelination and remyelination following injury (Santos and Fields, 2021). Our ongoing studies address the impact of the *Csf1r*^E631K/+^ genotype in various disease models.

Notwithstanding the lack of brain pathology, the data demonstrate that a heterozygous kinase-dead ALSP-associated mutation in the germline compromises CSF1R signalling. In light of the functional analysis (Pridans et al., 2013) and the lack of impact of heterozygous loss-of-function mutations in mouse, rat and human, we favour a dominant-negative mechanism for the predominant kinase-dead mutations in ALSP (Hume et al., 2020), although the possibility that a 50% loss of CSF1R function can lead to neuropathology is not excluded. The *Csf1r*^E631K/+^ mouse provides a hypomorphic model for understanding CSF1R biology and may also provide insight into the likely selective impacts of CSF1R kinase inhibitors on peripheral macrophage populations.

## MATERIALS AND METHODS

### CRISPR/Cas9 design

CRISPR/Cas9 was used to introduce into the mouse germline a mutation altering amino acid 631 in mouse CSF1R from glutamic acid to lysine (E631K). The methods used for targeted mutagenesis were similar to those previously used to insert a Fusion Red cassette into the mouse *Csf1r* locus (Grabert et al., 2020). Guides were designed using the Sanger website (http://www.sanger.ac.uk/htgt/wge/) with stringent criteria for off-target predictions [guides with mismatch (MM) of 1 or 2 for elsewhere in the genome were discounted]. Single-stranded RNA (ssRNA) guide 513 (g513) was used to cut the sequence around the glutamate codon at position 631 of *Csf1r* on chromosome 18. A ssDNA template then induced the base change GaG to AaA, converting the glutamate codon to a lysine codon and deleting the Alul site to allow for genotyping (Fig. S1A). Synthetic Alt-R crRNA and tcacrRNA and single-stranded DNA repair template oligonucleotides were obtained from Integrated DNA Technologies (Leuven, Belgium). The Alt-R crRNA oligo was resuspended in sterile RNase free injection buffer (Tris HCl 1 mM, pH 7.5, EDTA 0.1 mM) and annealed with tracrRNA by combining 2.5 μg crRNA with 5 μg tracrRNA and heating to 95°C. The mix was left to cool slowly to room temperature (RT). After annealing the complex, an equimolar amount was mixed with 1000 ng Cas9 recombinant protein (New England Biolabs, final concentration 20 ng/μl) and incubated at RT for 15 min before adding Cas9 mRNA (final concentration; 20 ng/μl) and the ssDNA PAGE purified repair template (final concentration 50 ng/μl) in a total injection buffer volume of 50 μl. The injection mix was centrifuged at 14,000 ***g*** for 10 min at RT and the top 40 μl then removed for microinjection.

### Generation of C57BL/6J.Csf1r^Em1Uman^ (Tg16) mice

Microinjections were performed using the AltR crRNA:tracrRNA:Cas9 complex (each at 20 ng/μl), Cas9 mRNA (20 ng/μl) and the ssDNA homology-directed repair (HDR) template (50 ng/μl). The mix was injected into 1-day-old single cell mouse embryos (C57BL/6JOlaHsd). The zygotes were cultured overnight, and the resulting two-cell embryos implanted into the oviduct of day-0.5 postcoitum pseudopregnant mice.

### Genotyping

Genotyping was performed using the Phire Direct Tissue PCR kit (Fisher Scientific, 15252606) using the storage and dilution protocol recommended by the manufacturer. Ear clips taken from mice at weaning were added to 20 μl of dilution buffer and 0.5 μl of DNA release and incubated at 98°C for 2 min. PCR was then performed at an annealing temperature of 62°C with forward (5′ACGCCTGCATTTCTCATTCC) and reverse primers (5′ATCCAGCTCTTACCTCCGTG). The DNA was then digested with 2 μl CutSmart Buffer (NEB, R0137L) and 1 μl Alul enzyme (NEB, R0137L) at 37°C for 1 h. The products were then separated on a 2% agarose gel. The expected products were 121 bp, 77, bp, 6 bp (+/+), 198 bp, 121 bp, 77 bp, 6 bp (+/E631K) and 198 bp, 6 bp (E631K/E631K) (Fig. S1B). For ongoing breeding, a quantitative (q)PCR-based protocol was also devised. Two qPCR reactions were run in parallel, using a forward primer specific for either WT *Csf1r* (WT-forward: AAGGAGGCCCTGATGTCAGAG) or *Csf1r-*E631K (MUT-forward: AAGGAGGCCCTGATGTCAAAA) with a universal reverse primer (reverse: ACAGGCTCCCAAGAGGTTGA). The WT *Csf1r* allele was poorly amplified using the mutant primer and vice versa (delta Ct ∼10). *Csf1r-*EGFP mice were genotyped by qPCR using GFP-specific primers (forward: ACTACAACAGCCACAACGTCTATATCA; reverse: GGCGGATCTTGAAGTTCACC).

### Animal breeding

C57BL/6J.Csf1r^Em1Uman^ (Tg16) donor and recipient mice were of a C57BL/6JOlaHsd background. They were then crossbred on a C57BL/6JCrl background with interbreeding of the offspring and then further backcrossed to C57BL/6JCrl mice. After the transfer from Edinburgh to Australia, the mice were rederived and bred and maintained in specific pathogen-free facilities at the University of Queensland facility within the Translational Research Institute, Brisbane. To enable visualisation of myeloid populations in tissues, the *Csf1r*^E631K^ line was bred to the *Csf1r*-EGFP reporter transgenic line (Sasmono et al., 2003) also backcrossed more than ten times to the C57BL/6JArc genetic background. For comparative analysis, mice bearing the *Csf1r*^ΔFIRE^ hypomorphic allele (Rojo et al., 2019) were transferred from Edinburgh to UC Irvine and bred to the C57BL/6J genetic background. For the analysis of embryos, timed matings were set up during the late afternoon. If a plug was detected the next morning, this was considered as E0.5

### Animal ethics

In the UK, ethical approval was obtained from The University of Edinburgh's Protocols and Ethics Committees under the authority of a UK Home Office Project Licence under the regulations of the Animals (Scientific Procedures) Act 1986. In Australia, all studies were approved by the Animal Ethics Committee of the University of Queensland. Mice were housed and bred under specific pathogen-free conditions in both locations.

### Tissue processing

Timed matings were set up in the late afternoon and mice were plug checked the next morning. If a plug was found this was considered as E0.5. Pregnant mothers were euthanised with carbon dioxide at the times indicated, uteri removed on ice and embryos removed. Embryos were fixed in 10% neutral buffered formalin for 2 days, transferred to 70% ethanol and then cut in half along the sagittal plane using a scalpel. For postnatal analyses, peripheral blood and peritoneal cells were collected as previously described (Rojo et al., 2019). Following peritoneal lavage, tissues of interest were removed. Tissue for qPCR analysis was snap frozen in TRI Reagent (Sigma, T9424). Tissues for immunohistochemistry (IHC) were post-fixed in 4% paraformaldehyde (PFA) for ∼6 h and then transferred to 1× PBS with 0.01% sodium azide. Tissues were embedded in paraffin using standard methods by core histology facilities at the Queen's Medical Research Institute, Edinburgh or the Translational Research Institute.

### Immunohistochemistry

Sources of antibodies and other reagents are provided in Table S1.

For IHC of embryos, antigen retrieval was performed with Vector Antigen Unmasking solution at 100°C for 5 min. Nonspecific protein binding was blocked with 2.5% horse serum for 20 min. For microglia detection, slides were incubated with rabbit anti-IBA1 primary antibodies (1:2000) at room temperature for 30 min. After washing in PBS, slides were incubated with secondary antibodies at RT for 35 min. Following two washes in PBS, slides were incubated with peroxidase substrate for 5 min. Slides were finally washed and counterstained with Haematoxylin and Eosin (H&E), then dehydrated before mounting with Pertex mounting medium (Pioneer Research, PRC/R/750).

Tissues for whole-mount imaging were extracted and kept in PBS on ice until imaged with an Olympus FV3000 confocal microscope. For IHC analysis of adults, tissues were fixed and processed for paraffin-embedded histology using routine methods, with the exception of 7-week-old brains, which were reserved for free-floating IHC. These brains were cryoprotected, frozen in OCT (ProSciTech, IA018) and 40-µm serial, coronal sections were collected in a rostrocaudal manner (1 in 12 series) using a Leica CM1950 cryostat. In addition, 7-week-old spleens were fixed, cryoprotected and then frozen in OCT.

Frozen spleen sections (5 μm) were sequentially stained with F4/80 and CD169 (Table S1). Free-floating brain sections were incubated at RT for 30 min in permeabilisation buffer (0.1% Triton X-100 in PBS) followed by 90 min in blocking solution (5% goat serum, 0.3% Triton X-100 in PBS). Sections were then incubated overnight at 4°C under orbital agitation in the primary antibodies against defined surface markers. Following 3×10 min washes in permeabilisation buffer, slices were incubated in the appropriate secondary antibodies (Table S1) diluted in blocking solution, for 90 min at RT in the dark. Slices were then washed in permeabilisation buffer, followed by a 5 min incubation with 4′,6-diamidino-2-phenylindole (DAPI) diluted in PBS, and a further 10 min wash in permeabilisation buffer. All sections were then washed with PBS for 5 min and mounted with Fluorescence Mounting Medium (Agilent Technologies, s302380-2). Images were acquired on an Olympus FV3000 confocal microscope.

GFAP^+^, P2RY12^+^ and TMEM119^+^ areas were quantified using ImageJ. The percentage area of positive staining was calculated using the ‘measure’ tool in ImageJ, following adjustment of the brain region-specific threshold, which was kept consistent for all mice. To calculate IBA1^+^ cell body size, maximum intensity projections (MIPs) were opened in Fiji v1.5 and the free drawing tool was used to measure the area of positive cells with a visible nucleus (20 cells per animal). For microglial arborisation, images were analysed as previously described (Patkar et al., 2021b).

Paraffin-embedded tissues were sectioned at 6 μm using a Leica RM2245 microtome. F4/80 and IBA1 staining was performed as previously described (Keshvari et al., 2021a). Whole-slide digital imaging was performed on a VS120 Olympus slide scanner. IBA1^+^ density was analysed in four different fields per sample. In the liver, F4/80-positive areas were quantified as a percentage using the ‘measure’ tool in ImageJ, following adjustment of the image threshold, which was kept consistent for all mice. To quantify the corpus callosum area identified by staining with Luxol Fast Blue (Acros Organics, 212171000) (Pridans et al., 2018) the free drawing tool in Fiji was used to measure the area of the cerebral hemisphere, and then the corpus callosum contained within that hemisphere. The area of the corpus callosum as a percentage of the total area of the cerebral hemisphere was then calculated.

### Embryo image acquisition and quantification

Images were acquired using the NanoZoomer (Hamamatsu) slide scanner at 40× magnification. Image analysis was performed with NDP.view software (Hamamatsu) and ImageJ. For the embryo analysis, images were exported in tiff format from the NDP.view files. In ImageJ, the perimeter of the developing brain was traced and the area measured. Colour threshold settings were used to remove the white gaps in the developing brain for an exact area of brain. Further colour threshold settings were applied to measure IBA1 staining and a percentage of IBA1 staining in the whole developing brain was then calculated per embryo. The same ImageJ protocol was followed to quantify IBA1 staining in embryo livers.

### Analysis of response to CSF1 *in vitro* and *in vivo*

BM cells were harvested and cultured in varying concentrations of recombinant human CSF1 as described previously (Rojo et al., 2019). After 7 days, 25 µg/ml of resazurin was added to each well. Plates were returned to the incubator for 1 h. Optical density was then measured on a plate reader (Omega Pherastar, BMG Labtech).

To assess the *in vivo* response to CSF1, mice were injected with a recombinant human-CSF1 mouse Fc conjugate (provided by Novartis; development and characterisation of this protein is described elsewhere; Keshvari et al., 2022). Six-week-old littermates received one injection per day of 5 mg/kg CSF1-Fc for 4 days between Zeitgeber time (ZT) 2 and ZT3.

### Micro-CT imaging and reconstruction

Left-hind limbs (LHL) were fixed in 4% PFA and subsequently transferred into PBS for high-resolution micro-CT scanning using Bruker's Skyscan 1272. X-ray settings were standardised to 70 kV and 142 μA and used a 0.5-mm aluminium X-ray filter. Each entire femur was scanned over 360° rotation in 0.8° rotational steps and the exposure time was set to 470 ms. Projections were acquired with nominal resolutions of 10 μm and each slice contained 1224×820 pixels. All X-ray projections were reconstructed using a modified back-projection reconstruction algorithm (NRecon 1.7.3.1 software-SkyScan, Bruker) to create cross-sectional images. Reconstruction parameters included ring artefact correction (2-6), beam hardening correction (40-50%) and misalignment correction. Reconstruction was performed in a blinded manner. 3D reconstructions were viewed using CTvox 3.3.0 (Bruker). Reconstructed images were analysed through CTAn 1.19 software (Bruker), which has inherent 2D and 3D analysis tools. All analyses were performed as per the updated guidelines for the assessment of bone density and microarchitecture *in vivo* using high-resolution peripheral quantitative CT (Whittier et al., 2020).

### Magnetic resonance imaging

Magnetic resonance imaging (MRI) was performed on a 7T horizontal bore Biospec AVANCE neo preclinical imaging system equipped with a 116 mm bore gradient insert (Bruker BioSpin; maximum gradient strength 660 mT/m). Mice were anaesthetised with 1.5-2% isoflurane (Zoetis) in oxygen/air (50/50, 1 L/min) and secured in a cradle (Rapid Biomedical). The respiration rate and rectal temperature were monitored (Model 1030 monitoring and gating system, Small Animal Instruments), with body temperature maintained at 37°C by a heat fan. An 86 mm quadrature volume coil (Bruker BioSpin) was used for transmission with signal reception by a two-channel phased-array mouse brain coil (Rapid Biomedical).

Scout images were taken to confirm correct positioning and the magnetic field was optimised using an automated 3D field mapping routine. For all subsequent sequences, the field of view was 19.2×19.2 mm and the slice thickness was 0.8 mm. For anatomical imaging, 17 coronal slices covering the entire brain were acquired using a T2-weighted Rapid Acquisition with Relaxation Enhancement (RARE) sequence with the following parameters: matrix size 192×192, TR 2300 ms, effective TE 36 ms, RARE factor 4, number of signal averages 4. The scan time was 7 min 21 s.

### Flow cytometry

Microglial cells were isolated as described by Grabert et al. (2020). Myeloid lineage, HSPC and CP subset phenotyping was performed on BM suspensions as detailed in Table S1. Peripheral blood, peritoneal lavage cells and isolated microglia were also stained for myeloid lineages as detailed in Table S1. Cell acquisition was performed on a Beckman Coulter Cytoflex Analyser or BD LSRFortessa™ X-20. Data analysis was performed using the FlowJo software (Tree Star Data Analysis Software).

### IGF1 and CSF1 immunoassay

Serum IGF1 and serum CSF1 were measured using commercial kits (Table S1) according to the manufacturer's instructions.

### RNA purification and qRT-PCR analysis

mRNA isolation, quantification and assessment of integrity were carried out as described previously (Keshvari et al., 2021a) and gene expression was quantified using the SYBR Select Master Mix on an Applied Biosystems QuantStudio real-time PCR system. Gene expression relative to *Tbp1* (*Psmc3*) was calculated using the ΔCt method supported by QuantStudio software.

The primer sequences used were: *Mmp9* forward: AGGGGCGTGTCTGGAGATTC; *Mmp9* reverse: TCCAGGGCACACCAGAGAAC; *Plau* forward: GGTTCGCAGCCATCTACCAG; *Plau* reverse: TTCCTTCTTTGGGAGTTGAATGAA; *Tbp1* forward: CTCAGTTACAGGTGGCAGCA; *Tbp1* reverse: ACCAACAATCACCAACAGCA.

### Sensorimotor testing

Sensorimotor testing was conducted on 43-week-old mice. In all tests, the experimenter was blinded as to the genotype of each mouse. The mechanical paw withdrawal threshold was measured using an electronic von Frey apparatus (MouseMet Electronic von Frey, Topcat Metrology). Mice were habituated in individual mouse runs for 30 min prior to commencing measurement. As described previously (Hasan et al., 2021), a soft-tipped von Frey filament was placed against the foot pad of the right hind paw. Pressure was slowly increased at a rate of 1 g/s, through rotation of the device handle, and the force (***g***) causing paw withdrawal displayed on the device was recorded. A single biological replicate was determined by averaging three repeated measurements (minimum of 5-min intervals) for each mouse (Hasan et al., 2021).

Locomotor performance was measured using the Parallel Rod Floor apparatus (Stoelting Co.). Each mouse was placed into the centre of the apparatus. The total distance travelled (m) and number of errors (foot slips off the rods) were recorded over a period of 2 mins and analysed using the ANY-Maze software (Stoelting Co.). Gait analysis was performed using CatWalk XT (Noldus Information Technology). Animals were allowed to voluntarily traverse the enclosed, illuminated glass surface. All recordings were performed in a dark room. A camera captured the illuminated footprints from below the glass surface to record the paw placement of each mouse as it traversed the platform. Only runs of 3-12 s duration with speed variances below 90% were considered acceptable. The mouse remained on the platform until three acceptable runs had been recorded. Analysis of these runs was performed using CatWalk XT software.

### Data analysis

Analysis of histological, flow cytometry, and behavioural data was performed blinded to genotype and treatment group. Data are presented as mean±s.d. Statistical tests were performed using GraphPad Prism 8.0.1. For data comparing baseline genotype differences, analysis was performed using unpaired Student’s *t*-tests. To compare the *in vitro* response to CSF1 and GM-CSF, a two-way ANOVA with Sidak’s multiple comparisons testing was used. To analyse differences in response to *in vivo* CSF1-Fc treatment, Mann-Whitney U tests were used. All tests were two-tailed.

## Supplementary Material

Supplementary information

Reviewer comments
