# Supplementary material for: A kinase-dead Csf1r mutation associated with adult-onset leukoencephalopathy has a dominant inhibitory impact on CSF1R signalling
Source: Development. 2022 Mar 25;149(8):dev200237. doi: 10.1242/dev.200237 (PMC9002114; doi:10.1242/dev.200237)
Supplement: Supplementary information [file develop-149-200237-s1.pdf]

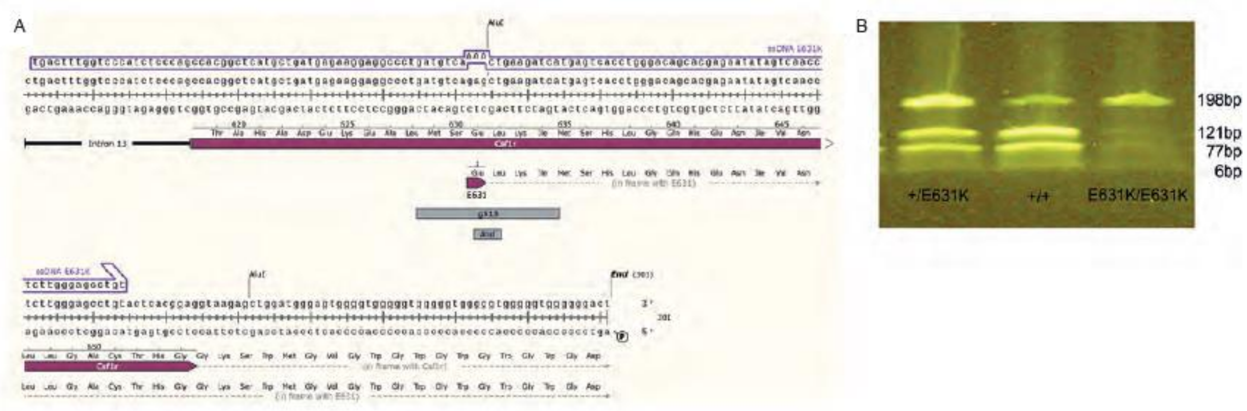

Fig. S1. Generation of the *Csf1r* E631K mutation and genotyping of the founders

(A) The sequence of mouse *Csf1r* genomic DNA and the single stranded RNA (ssRNA) guide 513 (g513) used to cut the sequence around the glutamate codon at position 631 of Csf1r on chromosome 18. The ssDNA template then induced the base change GaG to AaA converting to a lysine codon. This process also deleted an AluI site A(AGCT) which enabled genotyping. **(B)** The detection of +/+, +/E631K and E631K/E631K mice based upon Alu1 digestion of a PCR product spanning the mutation.

Development • Supplementary information

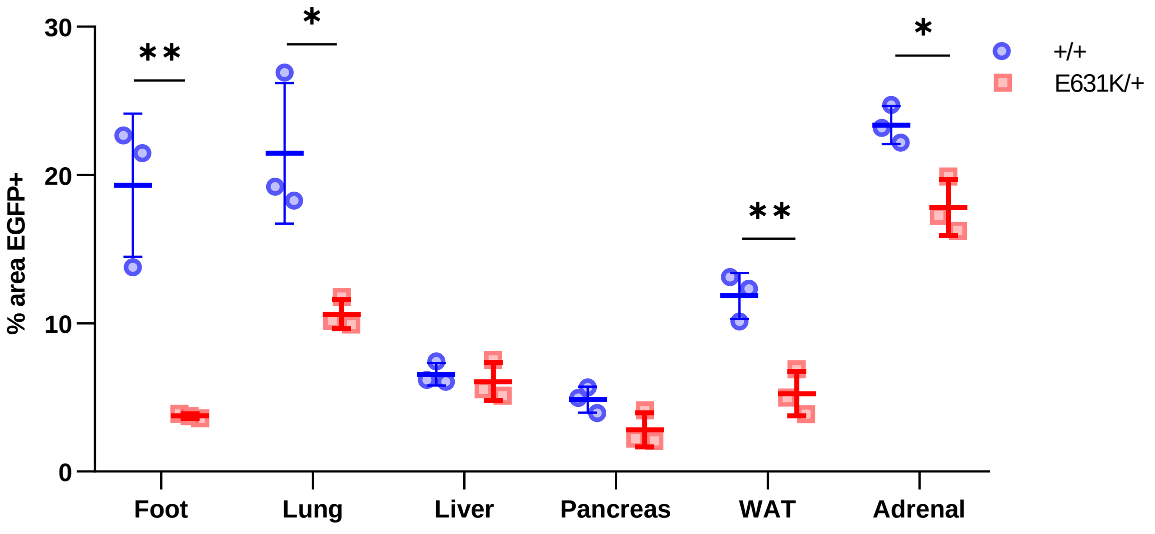

Fig. S2. Quantification of the effect of heterozygous Csf1r E631K mutation on tissue macrophages at 3 weeks of age

Tissues were extracted from 3-week-old male *Csf1r*$^{+/+}$ and *Csf1r*$^{E631K/+}$ littermates, each also *Csf1r*-EGFP transgenic. The tissues were placed in PBS on ice and imaged directly within 2–3 h using an Olympus FV3000 microscope. Maximum Intensity Projections were created from 10 consecutive Z-stacks and the % area positive for GFP was quantified in ImageJ. Individual data points represent the average of 3 areas from within the same image. Mean and standard deviation are presented. Statistical analysis was performed using a one-sided, unpaired student's t-test. *, ** = p<0.05, 0.01

Development • Supplementary information

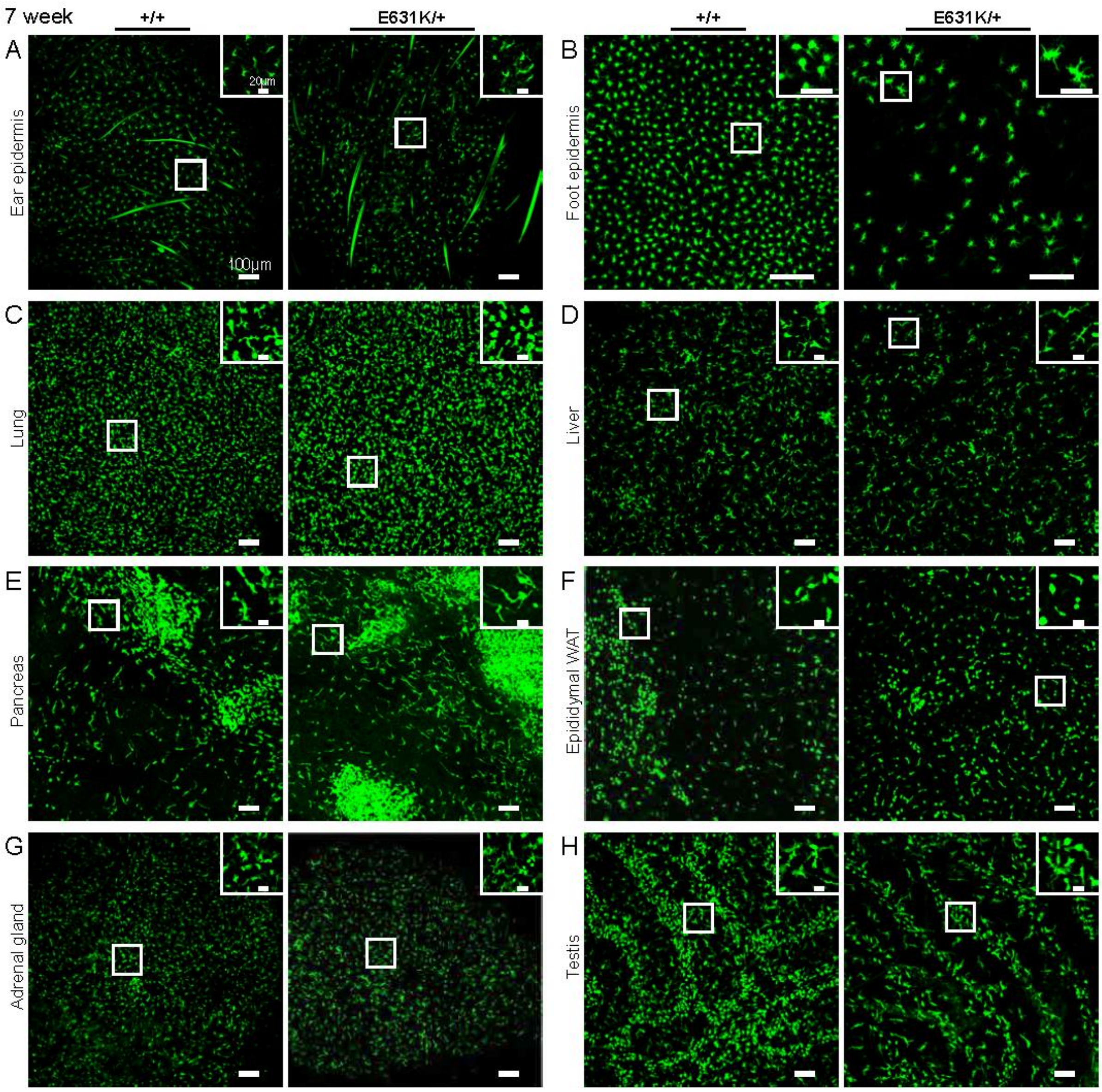

Fig. S3. The effect of heterozygous Csf1r E631K mutation of tissue macrophages at 7 weeks of age

Tissues were extracted from male Csf1r+/+ and Csf1rE631K/+ littermates, each also Csf1r-EGFP transgenic. The tissues were placed in PBS on ice and imaged directly within 2–3 h using an Olympus FV3000 microscope. **(A-H)** Images show the same depth of maximum intensity projections of the tissues indicated and are representative of at least 3 mice of each genotype. Note the large aggregates of Csf1r-EGFP+ cells on the surface of pancreas **(E)**.

Development • Supplementary information

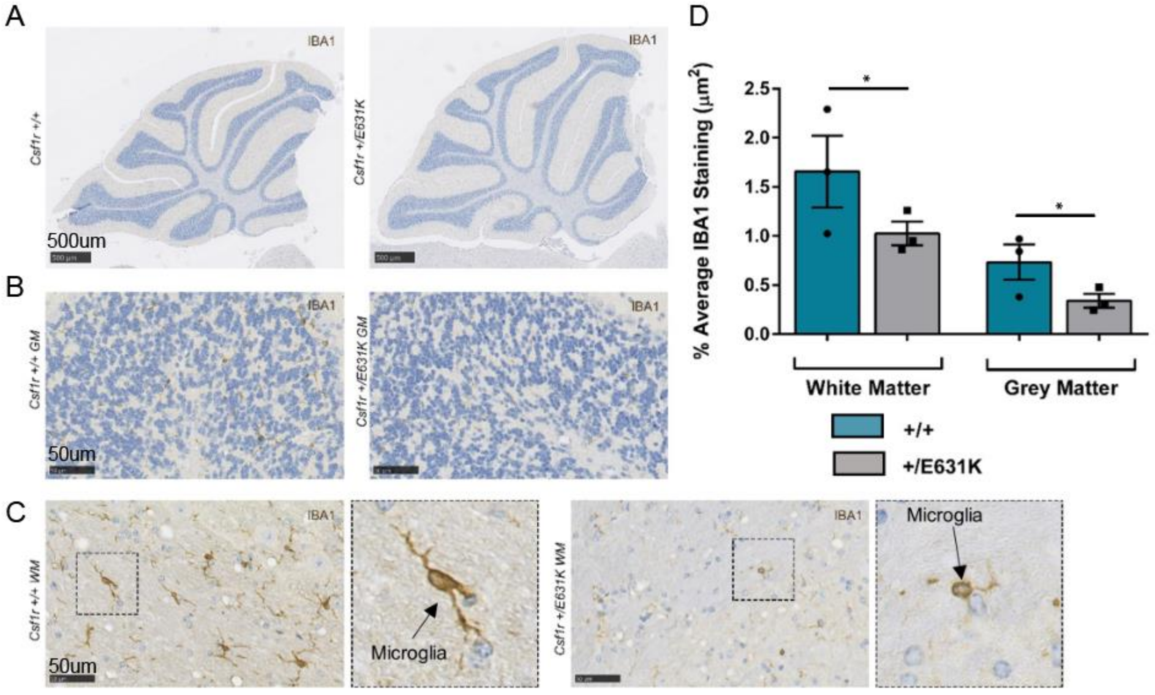

Fig. S4. The effect of heterozygous *Csf1r* E631K mutation on the cerebellum.

These data represent an independent analysis of the effect of the mutation carried out at the University of Edinburgh. Brains taken from 9-week-old mice were fixed, embedded and processed as described in Materials and Methods. The brains were the cerebellum was cut along the sagittal plane. **(A-C)** IBA1 staining was performed on cerebellar slices. Images were acquired using the NanoZoomer (Hamamatsu) slide scanner at 40X magnification. Image analysis was performed with NDP.view software (Hamamatsu) and ImageJ. **(B, C)** For the cerebellum 5 images at 40X magnification (440.35 μm x 249.53 μm) were taken of the grey matter (GM) and white matter (WM). Image J colour threshold settings were applied to quantify the average percentage of IBA1 staining. An overall average of the 4 brain slices was calculated per mouse. **(D)** The average percentage of IBA1+ staining in the cerebellar GM and WM. Individual data points with mean and standard deviation are presented. Statistical analysis was performed using unpaired student's t-test, * = $p<0.05$.

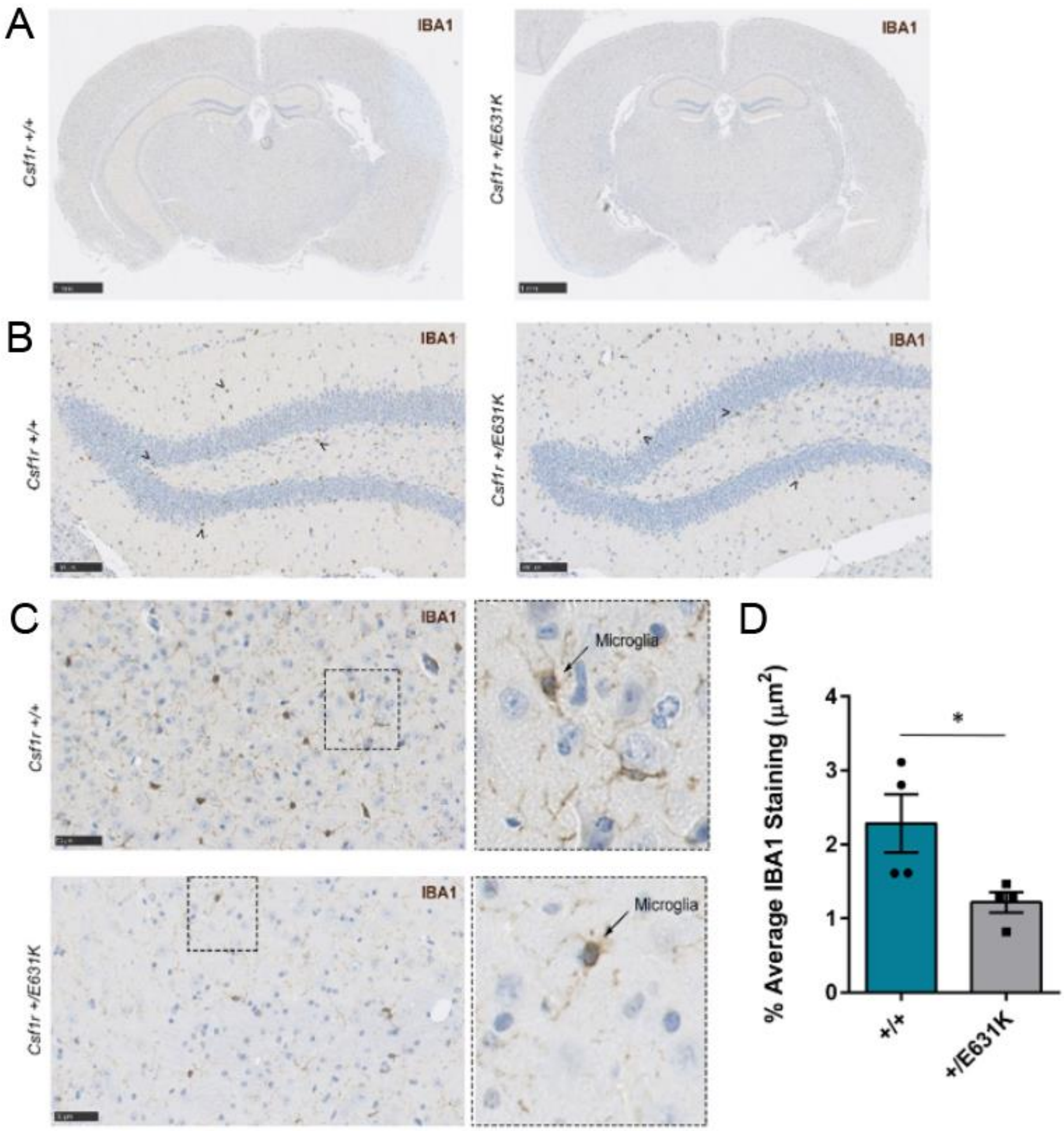

Fig. S5. The effect of heterozygous *Csf1r* E631K mutation on the hippocampus.

These data represent an independent analysis of the effect of the mutation carried out at the University of Edinburgh in the same animals as in Figure S3 and with the same methods. **(A-C)** Representative images of the brain, dentate gyrus and individual IBA1+ microglia in the two genotypes. **(D)** The % average of IBA1+ area was quantified. Individual data points with mean and standard deviation are presented. Statistical analysis was performed using unpaired student's t-test, * = $p<0.01$.

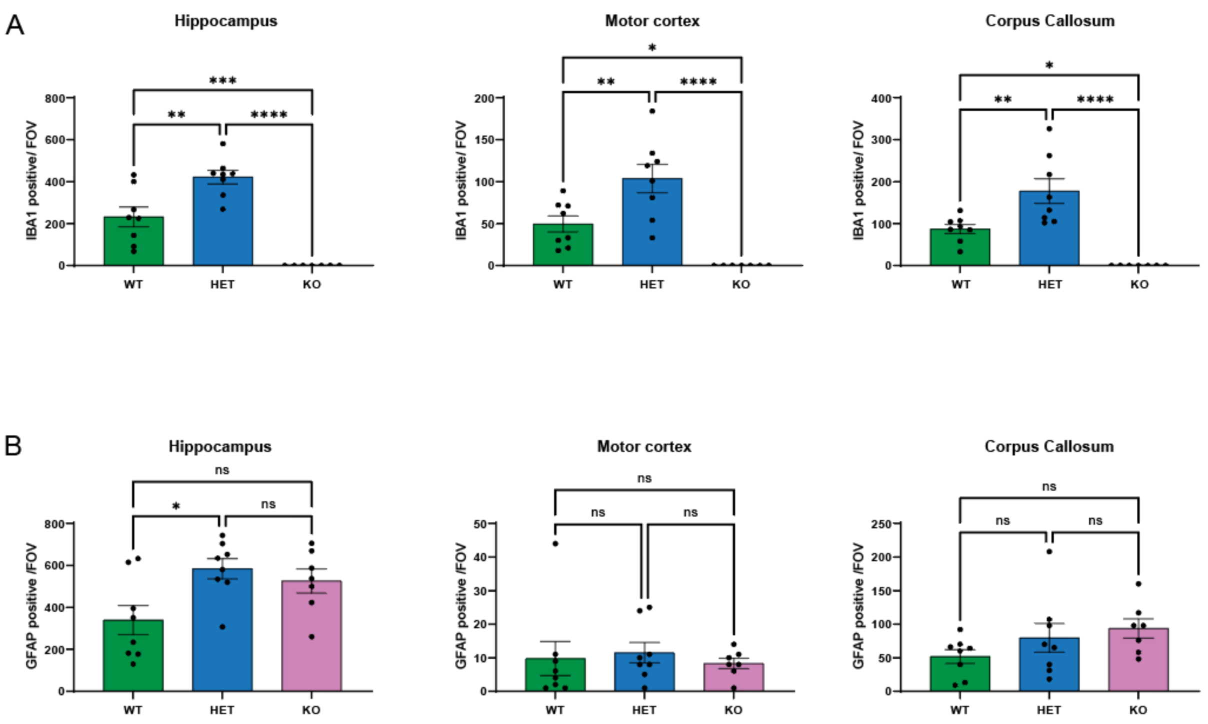

Fig. S6. The effect of *Csf1r*^{DFIRE} mutation on microglial density in aged mice.

(A) IBA1+ cell quantification in the hippocampus, motor cortex and corpus callosum. **(B)** GFAP+ cell quantification in the hippocampus, motor cortex, and corpus callosum. 6-month-old *Csf1r*$^{+/+}$ (WT) , *Csf1r*$^{DFIRE/+}$ (HET) and *Csf1r*$^{DFIRE/DFIRE}$ (HOM) (n=4 F/ n=4 M) housed at UC Irvine were processed for IHC. Brains were sectioned coronally into 30 µm-thick slices on a freezing microtome (Leica SM 2010R) and stained as free-floating slices. 40X confocal images were taken from hippocampus, motor cortex, and corpus callosum. IBA1 and GFAP positive cells were quantified using Imaris software (version 9.6.0).

Development • Supplementary information

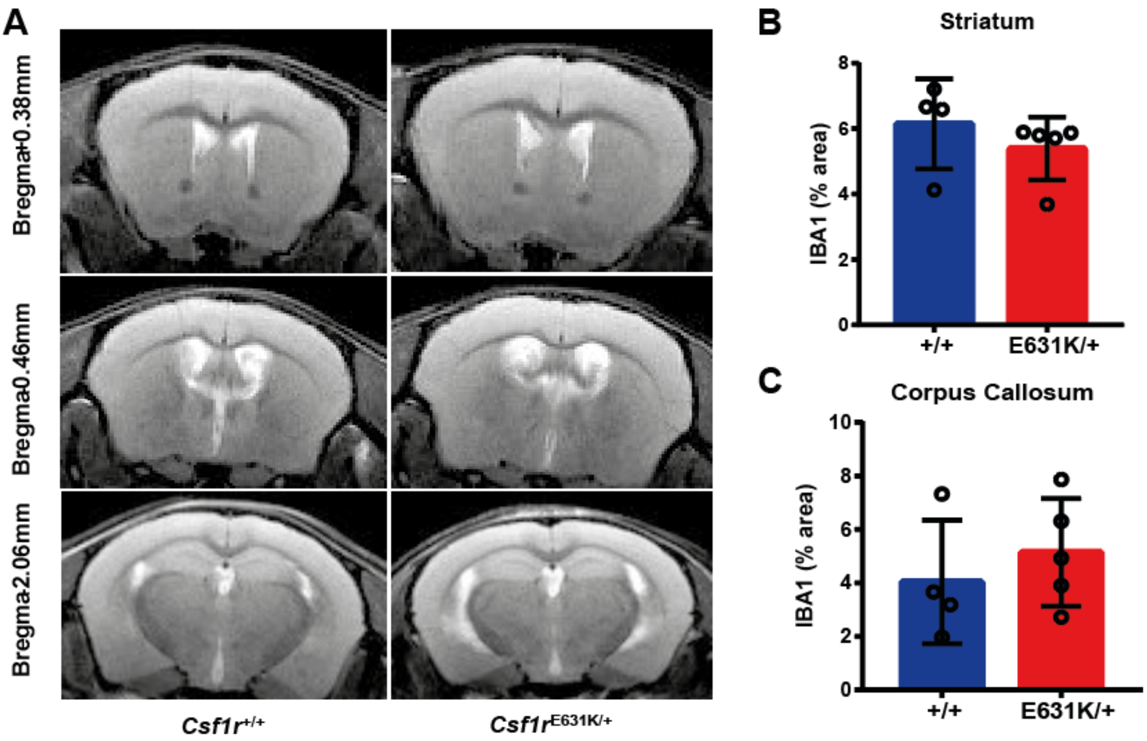

Fig. S7. The effect of heterozygous *Csf1r* E631K mutation on the brain in aged mice.

(A) Magnetic resonance imaging analysis of 15-month-old mice. Coronal structural T2-weighted scans of *Csf1r*$^{+/+}$ and *Csf1r*$^{E631K/+}$ mice at three levels relative to the Bregma as indicated. Scans are representative of n=5 for each genotype. Quantitative analysis of individual structures revealed no significant differences. **(B, C)** Relative density of IBA1 immunoreactivity in striatum and corpus callosum of 15-month-old mice. Brains were fixed and paraffin-embedded. Coronal sections were stained to detect IBA1^{+} microglia. Images of the striatum and corpus callosum were obtained on a Zeiss Axio Imager 2 at 100x magnification. IBA1 immunostaining was quantified using Image J software by converting images to 8-bit and applying thresholding to distinguish IBA1 from background and calculating the percentage area of staining. Individual data points with mean and standard deviation are presented in each graph (n=5/group). Statistical analysis was performed with an unpaired two-tailed Student's t-test with no statistically significant difference between genotypes.

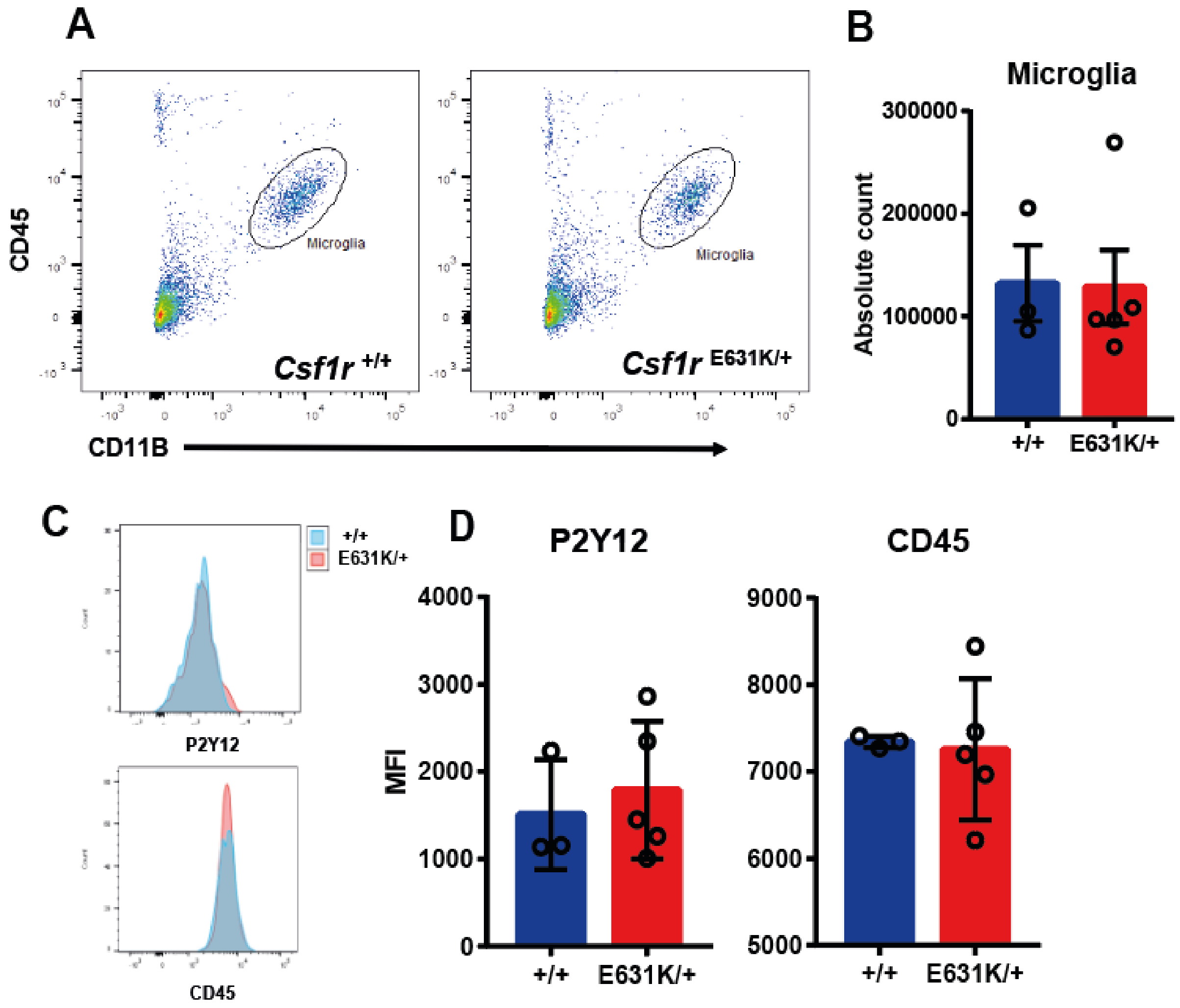

Fig. S8. The effect of heterozygous *Csf1r* E631K mutation on the brain in aged mice.

Brains of 15-month-old *Csf1r*$^{+/+}$ and *Csf1r*$^{E631K/+}$ mice were digested to release microglia as described in Materials and Methods and stained with direct conjugated antibodies against CD45, CD11b and P2RY12. **(A)** Representative FACS profiles identifying CD45^{lo}, CD11b^{hi} microglial cells. **(B)** The absolute numbers of CD45^{lo}, CD11b^{hi} microglia isolated per brain. **(C)** Representative histograms of P2RY12 and CD45 on this population. (D) The MFI for each of these markers on microglia from the 5 mice of each genotype. Statistical analysis was performed using unpaired student's t-test with no statistical significance between genotypes.

Table S1. Reagents List

Click here to download Table S1

Development • Supplementary information
