# Supplementary material for: A kinase-dead Csf1r mutation associated with adult-onset leukoencephalopathy has a dominant inhibitory impact on CSF1R signalling
Source: Development. 2022 Mar 25;149(8):dev200237. doi: 10.1242/dev.200237 (PMC9002114; doi:10.1242/dev.200237)
Supplement: Reviewer comments [file develop-149-200237_review_history.pdf]

A kinase-dead *Csf1r* mutation associated with adult-onset leukoencephalopathy has a dominant inhibitory impact on CSF1R signalling

Jennifer Stables, Emma K. Green, Anuj Sehgal, Omkar Patkar, Sahar Keshvari, Isis Taylor, Maisie E. Ashcroft, Kathleen Grabert, Evi Wollscheid-Lengeling, Stefan Szymkowiak, Barry W. McColl, Antony Adamson, Neil E. Humphreys, Werner Mueller, Hana Starobova, Irina Vetter, Sepideh Kiani Shabestari, Matthew M. Blurton-Jones, Kim M. Summers, Katharine M. Irvine, Clare Pridans and David A. Hume
DOI: 10.1242/dev.200237

Editor: Florent Ginhoux

Review timeline

Original submission:	29 September 2021
Editorial decision:	6 December 2021
First revision received:	18 January 2022
Accepted:	4 February 2022

Original submission

First decision letter

MS ID#: DEVELOP/2021/200237

MS TITLE: A kinase-dead Csf1r mutation associated with adult-onset leukoencephalopathy has a dominant-negative impact on CSF1R signaling.

AUTHORS: Jennifer Stables, Emma K Green, Anuj Sehgal, Omkar Patkar, Sahar Keshvari, Isis Taylor, Maisie E Ashcroft, Kathleen Grabert, Evi Wollscheid-Lengeling, Stefan Szymkowiak, Barry A McColl, Antony Adamson, Neil Humphreys, Werner Mueller, Hana Starobova, Irina Vetter, Sepideh Kiani Shabestari, Mathew Blurton-Jones, Kim M Summers, Katharine M Irvine, Clare Pridans, and David A Hume

I have now received all the referees' reports on the above manuscript, and have reached a decision. The referees' comments are appended below, or you can access them online: please go to BenchPress and click on the 'Manuscripts with Decisions' queue in the Author Area.

As you will see, the referees express considerable interest in your work, but have some significant criticisms and recommend a substantial revision of your manuscript before we can consider publication. If you are able to revise the manuscript along the lines suggested, which may involve further experiments, I will be happy receive a revised version of the manuscript. Your revised paper will be re-reviewed by one or more of the original referees, and acceptance of your manuscript will depend on your addressing satisfactorily the reviewers' major concerns. Please also note that Development will normally permit only one round of major revision.

We are aware that you may be experiencing disruption to the normal running of your lab that make experimental revisions challenging. If it would be helpful, we encourage you to contact us to discuss your revision in greater detail. Please send us a point-by-point response indicating where you are able to address concerns raised (either experimentally or by changes to the text) and where you will not be able to do so within the normal timeframe of a revision. We will then provide further guidance. Please also note that we are happy to extend revision timeframes as necessary.

© 2022. Published by The Company of Biologists under the terms of the Creative Commons Attribution License (https://creativecommons.org/licenses/by/4.0/).

Please attend to all of the reviewers' comments and ensure that you clearly highlight all changes made in the revised manuscript. Please avoid using 'Tracked changes' in Word files as these are lost in PDF conversion. I should be grateful if you would also provide a point-by-point response detailing how you have dealt with the points raised by the reviewers in the 'Response to Reviewers' box. If you do not agree with any of their criticisms or suggestions please explain clearly why this is so.

Reviewer 1

Advance summary and potential significance to field

Stables et al. characterize a new mouse model carrying a mutation within the Csf1r gene that is found in ALSP patients. Using histology, CT, transgenic mouse models and behavioural tests, they show that mice homozygous for the mutation phenocopy the full Csf1r KO. However, heterozygous animals lack responsiveness to CSF1 stimulation, indicating that the mutation has a dominant-negative impact on Csf1r signalling. Albeit the phenotype of the mouse model resembles ALSP to some extent, a behavioural phenotype in motor function is lacking.

In summary, this is a very extensive characterization of the ALSP mouse model which gives insights into underlying mechanisms of leukoencephalopathy associated diseases.

Comments for the author

Few questions remain unanswered, which could provide an even better understanding of Csf1r signalling and the different models analysed in the manuscript.
1. Do macrophages in tissues analyzed by histology (e.g. Figure 3) have a different immunological phenotype in the E631K/+ model? Are these cells then monocyte-derived in all the tissues? Usage of more surface markers could at least indicate the origin of these cells.
2. The authors write that they were not able to quantify CD115 expression due to the autofluorescence of cells. This is a common problem when working with macrophages. Thus, the authors should use a fluorochrome that would allow circumventing this technical problem.
3. Colocalization of TMEM119 and P2RY12 is quantified, but from the pictures shown in Figure 6H, it is impossible to see this effect. Insets of the IF here and also in other subfigures, would help the reader to see these effects as well.
4. Sometimes the authors cite overview works instead of original papers (e.g. for function of microglia). Please cite original works as much as possible.
5. The comparison of the E631K with FIRE mice is interesting. However different time points were analyzed and are therefore not really comparable.
Please include additional time points.

Reviewer 2

Advance summary and potential significance to field

ALSP (previously called HDLS) is an autosomal dominant, adult onset neurodegenerative disease caused by mutations in CSF1R. It is especially interesting for macrophage and CSF1R biologists because is thought to be an example of a true "microgliopathy" where microglial dysfunction is a primary cause of disease. Stables and colleagues here provide the first characterization of their mouse model for ALSP, which was created by knocking in the mouse ortholog (E631K) of a human ALSP causing variant into the mouse Csf1r locus. They provide in vivo evidence that when homozygous, E631K phenocopies the Csf1r KO mouse, but when heterozygous, seems to have more severe developmental and homeostatic effects on macrophages than would likely be explained by haploinsufficiency: reduced tissue macrophage numbers early in life (and persistently in microglia), lower expression of CSF1R on myeloid lineage cells, and a blunted response to CSF1 ligand administration (as CSF1-Fc fusion protein). Importantly, they find no effects of E631K/+ on mechanical allodynia or motor function unlike in ALSP patients.

These findings are particularly important because another proposed ALSP model is the CSF1R +/- mouse, which has features of a leukodystrophy late in life, but seems to have increased numbers of

© 2022. Published by The Company of Biologists under the terms of the Creative Commons Attribution License (https://creativecommons.org/licenses/by/4.0/).

microglia rather than decreased as is found in human patients and in the Stables model here, and so may not reflect the same pathophysiology as ALSP. Based on their findings, the authors conclude that 1) the CSF1R +/- model is unlikely to be valid for ALSP and 2) that their findings in vivo prove that E631K has a dominant negative effect on CSF1R signaling.

Comments for the author

I have some concerns with the authors' interpretation of their data (see below), but overall this is an important contribution that should be published as it contributes to clarifying the mechanisms underlying ALSP, and offers a powerful model for future study. As written, there are two major and related issues that limit its impact:
1. Dominant negative effects: The authors conclude that E631K has dominant neg effects on CSF1R signaling. This is an important point because it would help to explain why ALSP is typically autosomal dominant. Prior studies, to my knowledge, have also not effectively tested whether ALSP mutations are dominant negative, though indirectly argue for (Pridans 2013 from this paper's ref list) and against (Konno 2014) at DN effect of ALSP causing mutations. The data shown here are particularly strong as they are in vivo, however do not directly test whether E631K is exerting a dominant negative (DN) effect on CSF1R signaling.
There are many "proxy" readouts for Csf1r signaling being very compromised in E631K/+ compared to +/+, particularly the CSF1-Fc administration experiments and observation of reduced tissue macrophage density. These studies argue that E631K could (or even likely does) exert a DN effect on Csf1r signaling, but no experiments show that E631K reduces CSF1R signaling more significantly than haploinsufficiency, or prove at a molecular level the DN effect. This would ideally be addressed experimentally (and the authors now have a source to easily get primary cells to test), but could also be addressed by carefully adjusting the claims made to reflect the experiments performed.
2. No direct comparisons to CSF1R+/-: a central argument in this paper is that E631K/+ has different (and in many instances more severe) phenotypes than Csf1r+/- mice, however it lacks Csf1r+/- as a control condition. For some findings, particularly the clear difference in microglial density (more in Csf1r +/-, less in E631K/+) and behavioral phenotypes, this control can be determined "in silico" (eg, reading the papers and comparing findings between the manuscripts), but in many cases the data shows that E631K/+ has impaired Csf1r signaling, but there is no way to know from the experiments performed if this is "worse" than Csf1r +/-. This would ideally be addressed experimentally by comparing to Csf1r +/- (which could also help to address major point 1), or other more creative approaches. But given the value of this experimental model to the field and time it could take to do the above, careful editing of text and discussion to clarify this limitation is also appropriate.

Other points:
1. I think that this mouse was made by knocking in the mouse ortholog of a human disease causing ALSP variant, but it is difficult to appreciate this from the text. This is a key point because mouse Csf1 would be unlikely to signal through a human CSF1R variant, and could benefit from further clarification in the manuscript text.
2. Fig 3 (and associated supp) shows only representative images, would benefit from quantification
3. Fig 5A-F: It would be useful to also know if relative numbers of progenitor populatiosn are affected by E631K (not just CSF1R MFI), as is shown for peritoneal macs.
4. Fig 6H - the blue is really hard to see. And, what's the purple panel? It looks identical to the P2RY12 panels.
5. 6K - are the headings reversed? What age are these? In the rep image E631K/+ seems to have more GFAP staining, rather than less as shown in the quantification in L.

© 2022. Published by The Company of Biologists under the terms of the Creative Commons Attribution License (https://creativecommons.org/licenses/by/4.0/).

First revision

Author response to reviewers' comments

We thank both reviewers for a careful and thorough review.
Our point-by-point response to the reviewers is provided below.

Reviewer 1 Advance Summary and Potential Significance to Field:

Stables et al. characterize a new mouse model carrying a mutation within the Csf1r gene that is found in ALSP patients. Using histology, CT, transgenic mouse models, and behavioural tests, they show that mice homozygous for the mutation phenocopy the full Csf1r KO. However, heterozygous animals lack responsiveness to CSF1 stimulation, indicating that the mutation has a dominant-negative impact on Csf1r signalling. Albeit the phenotype of the mouse model resembles ALSP to some extent, a behavioural phenotype in motor function is lacking.

In summary, this is a very extensive characterization of the ALSP mouse model, which gives insights into underlying mechanisms of leukoencephalopathy associated diseases.

Reviewer 1 Comments for the Author:
Few questions remain unanswered, which could provide an even better understanding of Csf1r signalling and the different models analysed in the manuscript.

1. Do macrophages in tissues analyzed by histology (e.g. Figure 3) have a different immunological phenotype in the E631K/+ model? Are these cells then monocyte-derived in all the tissues? Usage of more surface markers could at least indicate the origin of these cells.

The rationale for our analysis of postnatal expansion of tissue macrophages as outlined in the original text has been modified to include specific reference to current views on ontogeny. *Macrophage populations of the mouse and rat expand substantially in the postnatal period. Organs grow rapidly and macrophage density in each organ also increases as evident from the increase in relative expression of macrophage-expressed transcripts including Csf1r {Summers, 2017}.* The relative contribution of local proliferation of macrophages seeded during embryonic development and postnatal monocyte infiltration varies amongst individual organs {Yona 2013, Ginhoux, 2016, Hume, 2019, Guilliams, 2020}. *The postnatal expansion of the resident mononuclear phagocyte populations is associated with a postnatal increase in Csf1 mRNA in most organs and is CSF1R-dependent…..We predicted that a dominant effect of the Csf1r-E631K allele on CSF1 responsiveness would compromise and delay this postnatal resident tissue macrophage expansion.*

The original text contained a remark that “residual cells appeared round and monocyte-like suggesting population of the niche was ongoing”. We have removed that comment from the manuscript. Ontogeny is not the focus of our study. The inference in this remark is not actually directly addressed by our data. We have no reason to suggest that the mutation does anything other than delay the postnatal CSF1-dependent expansion of tissue macrophages. Proper dissection of the ontogeny of macrophages in our model (e.g. whether the CSF1R mutation changes the relative contribution of monocytes) would require the combination of the mutant allele with a fate-mapping strategy (for example crossing the mutation to the Ms4a3 monocyte reporter transgene (PMID: 31491389)). We feel such an experiment addresses a different issue and is beyond the scope of the current study. Regardless of ontogeny, it is not controversial to state that the postnatal expansion of tissue macrophages is CSF1/CSF1R-dependent and our data conclusively demonstrate that the CSF1R E631K heterozygous mutation compromises that expansion.

With respect to the use of surface markers to address the origin of cells, we are not aware of any marker that provides a definitive indication of the developmental origin of tissue macrophages. Schulz et al. (PMID:22442384) claimed that high F4/80 is associated with cells of yolk sac/embryonic origin. However, subsequent studies showed, for example, that F4/80(hi) peritoneal macrophages are slowly replaced by BM-derived cells (PMID: 27292029). We do show

© 2022. Published by The Company of Biologists under the terms of the Creative Commons Attribution License (https://creativecommons.org/licenses/by/4.0/).

that despite the reduced CD115 in the large peritoneal macrophages, their relative abundance and the level of F4/80 is unaffected (Figure 5). The data in Figure 8 show that relative abundance of F4/80(hi) cells in liver and spleen in adults is unaffected by the mutation, and also demonstrates the selective loss and unique CSF1 responsiveness of CD169+ cells in spleen.

2. The authors write that they were not able to quantify CD115 expression due to the autofluorescence of cells. This is a common problem when working with macrophages. Thus, the authors should use a fluorochrome that would allow circumventing this technical problem.

We thank the reviewer for bringing this to our attention. This was an error. There is in fact minimal autofluorescence in the PE-Cy7 channel. The reviewer's comment led us to identify a technical issue with the FMO histogram in the original figure. CD115 staining is in fact well-separated from background in peritoneal macrophages as in all of our previous studies. We have substituted the representative histogram in Figure 5J to correct this error. The error did not affect the quantitative CD115 MFI data in Figure 5H-I and the conclusion is unchanged. The text has been modified to reflect the data.

3. Colocalization of TMEM119 and P2RY12 is quantified, but from the pictures shown in Figure 6H, it is impossible to see this effect. Insets of the IF here, and also in other subfigures, would help the reader to see these effects as well.

The images have been pseudo-coloured to make the contrast clearer, insets have been added and the legend has been corrected to make it clear exactly what has been shown.

4.Sometimes the authors cite overview works instead of original papers (e.g. for function of microglia). Please cite original works as much as possible.

We understand the concern that original work is submerged by exclusive citation of reviews. The reviewer will appreciate that there is a massive literature on the function of microglia in development and pathology. It would be difficult to choose a subset of original papers for citation without offending many other authors. Against that background, our description of a mouse *Csf1r*$^{\Delta FIRE}$ in which microglia are entirely absent was a major surprise to many. We have added some of the key source references and additional reviews to the introduction and discussion and in select places in results.

5.The comparison of the E631K with FIRE mice is interesting. However, different time points were analyzed and are therefore not really comparable. Please include additional time points.

We are not in a position to repeat a time course of the effect of the *Csf1r*$^{\Delta FIRE/+}$ mutation on microglial abundance. We agree that a more complete time course would be potentially interesting, but we do not feel it would alter the conclusion. At the 6 month time point we analysed we were able to reproduce the microgliosis reported in *Csf1r*$^{+/-}$ mice on the C57Bl/6J genetic background at a similar time point. A time course would be essential only had we failed to reproduce the effect. We have modified the text to make the purpose clearer and to emphasise the importance of genetic background.

Reviewer 2 Advance Summary and Potential Significance to Field:
ALSP (previously called HDLS) is an autosomal dominant, adult onset neurodegenerative disease caused by mutations in CSF1R. It is especially interesting for macrophage and CSF1R biologists because is thought to be an example of a true "microgliopathy" where microglial dysfunction is a primary cause of disease. Stables and colleagues here provide the first characterization of their mouse model for ALSP, which was created by knocking in the mouse ortholog (E631K) of a human ALSP causing variant into the mouse Csf1r locus. They provide in vivo evidence that when homozygous, E631K phenocopies the Csf1r KO mouse, but when heterozygous, seems to have more severe developmental and homeostatic effects on macrophages than would likely be explained by haploinsufficiency: reduced tissue macrophage numbers early in life (and persistently in microglia), lower expression of CSF1R on myeloid lineage cells, and a blunted response to CSF1 ligand administration (as CSF1-Fc fusion protein). Importantly, they find no effects of E631K/+ on mechanical allodynia or motor function, unlike in ALSP patients.

© 2022. Published by The Company of Biologists under the terms of the Creative Commons Attribution License (https://creativecommons.org/licenses/by/4.0/).

These findings are particularly important because another proposed ALSP model is the CSF1R +/- mouse, which has features of a leukodystrophy late in life, but seems to have increased numbers of microglia rather than decreased as is found in human patients and in the Stables model here, and so may not reflect the same pathophysiology as ALSP. Based on their findings, the authors conclude that 1) the CSF1R +/- model is unlikely to be valid for ALSP and 2) that their findings in vivo prove that E631K has a dominant negative effect on CSF1R signaling.

I have some concerns with the authors' interpretation of their data (see below), but overall this is an important contribution that should be published as it contributes to clarifying the mechanisms underlying ALSP, and offers a powerful model for future study. As written, there are two major and related issues that limit its impact:

1. Dominant negative effects: The authors conclude that E631K has dominant neg effects on CSF1R signaling. This is an important point because it would help to explain why ALSP is typically autosomal dominant. Prior studies, to my knowledge, have also not effectively tested whether ALSP mutations are dominant negative, though indirectly argue for (Pridans 2013 from this paper's ref list) and against (Konno 2014) at DN effect of ALSP causing mutations. The data shown here are particularly strong as they are in vivo, however do not directly test whether E631K is exerting a dominant negative (DN) effect on CSF1R signaling. There are many "proxy" readouts for Csf1r signaling being very compromised in E631K/+ compared to +/+, particularly the CSF1-Fc administration experiments and observation of reduced tissue macrophage density. These studies argue that E631K could (or even likely does) exert a DN effect on Csf1r signaling, but no experiments show that E631K reduces CSF1R signaling more significantly than haploinsufficiency, or prove at a molecular level the DN effect. This would ideally be addressed experimentally (and the authors now have a source to easily get primary cells to test), but could also be addressed by carefully adjusting the claims made to reflect the experiments performed.

This is a valid concern and we accept that we have not conclusively shown a dominant negative effect in the strictest sense that the mutant protein interacts with the wild-type protein to inhibit signalling (in effect, a gain-of-function mutation). That would require direct demonstration that the mutant and wild-type proteins are expressed at similar levels and bind to each other in primary cells, which is currently not feasible. What we have already shown is that kinase-dead mutant proteins can traffic to the cell surface and can bind CSF1 when expressed in a factor-dependent cell line (Pridans et al. (2013)). We are aware of the conflicting data of Konno et al. (2014). These authors transiently transfected mutant CSF1R expression plasmids in an HEK293 cell line that was already stably expressing wild-type CSF1R. They provided no evidence of transient transfection efficiency (indeed the transfection method is not documented at all). It is not clear that all of the transiently-transfected cells expressed both WT and mutant proteins. We have added an explicit comment on this study in the introduction.
To take account of the uncertainty about mechanism, we have replaced the phrase dominant-negative in the title and in most places in the text and carefully adjusted the claims as suggested. It is accurate, regardless of mechanism, to state that the effect of the mutation is dominant in the genetic sense in that there is a clear heterozygous phenotype. The ALSP disease is referred to as autosomal dominant in human patients. So, we have retained that wording. That said, as the reviewer recognises, we do have compelling proxy evidence supporting a dominant negative effect on CSF1R signalling. The dominant inhibitory effect of the kinase-dead mutation on BM cell response to CSF1 is demonstrated *in vitro* in Figure 8A and contrasts to the lack of effect of heterozygous loss of CSF1R in bone marrow in the FIRE mutant that was demonstrated previously and now cited (see also response to point 2).

2. No direct comparisons to CSF1R+/-: a central argument in this paper is that E631K/+ has different (and in many instances more severe) phenotypes than Csf1r+/- mice, however it lacks Csf1r+/- as a control condition. For some findings, particularly the clear difference in microglial density (more in Csf1r +/-, less in E631K/+) and behavioral phenotypes, this control can be determined "in silico" (eg, reading the papers and comparing findings between the manuscripts), but in many cases the data shows that E631K/+ has impaired Csf1r signaling, but there is no way to know from the experiments performed if this is "worse" than Csf1r +/-. This would ideally be addressed experimentally by comparing to Csf1r +/- (which could also help to address major point 1), or other more creative approaches. But given the value of this experimental model to the field

© 2022. Published by The Company of Biologists under the terms of the Creative Commons Attribution License (https://creativecommons.org/licenses/by/4.0/).

and time it could take to do the above, careful editing of text and discussion to clarify this limitation is also appropriate.

We greatly appreciate the reasonable and supportive nature of this comment. We do not have the *Csf1rko* allele in Australia and it would currently not be straightforward to obtain it (since many airlines are currently not transporting experimental animals).
We have inserted the following comment in the discussion.
The Csf1r$^{E631K/+}$ mice are clearly different from the reported phenotype of Csf1r$^{+/-}$ mice {Chitu, 2020;Chitu, 2015;Chitu, 2021} although the effects on peripheral macrophage populations and CSF1 responsiveness have not been analysed in the haploinsufficiency model. Without a direct comparison on the same genetic background we cannot conclude that the effect of the heterozygous kinase dead mutation is greater than the effect of a null mutation…….
That said, in addition to the heterozygous FIRE mutation in mice, we have also published a very extensive characterisation of the *Csf1r*$^{+/-}$ rat that has no neurodegenerative phenotype and as we note in the text, there are many examples of aged obligate human carriers of null CSF1R mutations who have not developed neuropathology. It is also notable that there was no reported evidence of haploinsufficiency in *Csf1r*$^{+/-}$ mice on an outbred background (PMID: 11756160). The mechanisms underlying the selective impact of the C57BL/6J background on penetrance of *Csf1r* mutations have not yet been analysed. Arguably the evidence of dominant negative effects of CSF1R mutations has been provided in humans; the vast majority of affected individuals with ALSP have coding mutations in the conserved amino acids in the kinase domain. As we have reviewed elsewhere, it is not evident that the very small number of examples of "haploinsufficiency" (i.e. heterozygous null alleles) leading to ALSP do not alter the function/expression of the wild-type allele (or indeed, that they are even causal). We have also now pointed out in the revised introduction that there is an obvious parallel in the differential effects of loss-of-function (haploinsufficiency) and kinase mutations in the closely-related KIT gene in mice and humans.

Other points:

1. I think that this mouse was made by knocking in the mouse ortholog of a human disease causing ALSP variant, but it is difficult to appreciate this from the text. This is a key point because mouse Csf1 would be unlikely to signal through a human CSF1R variant, and could benefit from further clarification in the manuscript text.

This has now been clarified in the introduction. E631 in mice is equivalent to E633 in humans, and the lysine substitution (E633K) has been found in multiple affected individuals in a multi-generational family. We have added a citation of the original description of the human mutation to the introduction.

2. Fig 3 (and associated supp) shows only representative images, would benefit from quantification

This has been done and a supplementary figure has been added

3. Fig 5A-F: It would be useful to also know if relative numbers of progenitor populations are affected by E631K (not just CSF1R MFI), as is shown for peritoneal macs.

There was no significant difference in total number of cells or the proportional quantification of each subset. Relative percentages of each population have been inserted on the FACS profiles.

4. Fig 6H - the blue is really hard to see. And, what's the purple panel? It looks identical to the P2RY12 panels.

The colours have been altered to highlight the overlap and the legend has been edited to make it clear what is being shown.

5. 6K - are the headings reversed? What age are these? In the rep image, E631K/+ seems to have more GFAP staining, rather than less as shown in the quantification in L.

© 2022. Published by The Company of Biologists under the terms of the Creative Commons Attribution License (https://creativecommons.org/licenses/by/4.0/).

We apologise for this labelling error which has been corrected. The panels have been reordered, the legend and the text in results has been corrected. We have also corrected an oversight in that panels 6N and 6O were not mentioned in the main text.

Second decision letter

MS ID#: DEVELOP/2021/200237

MS TITLE: A kinase-dead Csf1r mutation associated with adult-onset leukoencephalopathy has a dominant inhibitory impact on CSF1R signaling.

AUTHORS: Jennifer Stables, Emma K Green, Anuj Sehgal, Omkar Patkar, Sahar Keshvari, Isis Taylor, Maisie E Ashcroft, Kathleen Grabert, Evi Wollscheid-Lengeling, Stefan Szymkowiak, Barry A McColl, Antony Adamson, Neil Humphreys, Werner Mueller, Hana Starobova, Irina Vetter, Sepideh Kiani Shabestari, Mathew Blurton-Jones, Kim M Summers, Katharine M Irvine, Clare Pridans, and David A Hume
ARTICLE TYPE: Research Article

I am happy to tell you that your manuscript has been accepted for publication in Development, pending our standard ethics checks.

Reviewer 1

Advance summary and potential significance to field

All points have been addressed by changing figures or text passages.

Comments for the author

All points have been addressed by changing figures or text passages.

Reviewer 2

Advance summary and potential significance to field

As in initial review

Comments for the author

I think the authors have addressed the concerns raised successfully and would reccommend publication.

© 2022. Published by The Company of Biologists under the terms of the Creative Commons Attribution License (https://creativecommons.org/licenses/by/4.0/).
